# Multi-omic signature of body weight change: results from a population-based cohort study

**DOI:** 10.1186/s12916-015-0282-y

**Published:** 2015-03-09

**Authors:** Simone Wahl, Susanne Vogt, Ferdinand Stückler, Jan Krumsiek, Jörg Bartel, Tim Kacprowski, Katharina Schramm, Maren Carstensen, Wolfgang Rathmann, Michael Roden, Carolin Jourdan, Antti J Kangas, Pasi Soininen, Mika Ala-Korpela, Ute Nöthlings, Heiner Boeing, Fabian J Theis, Christa Meisinger, Melanie Waldenberger, Karsten Suhre, Georg Homuth, Christian Gieger, Gabi Kastenmüller, Thomas Illig, Jakob Linseisen, Annette Peters, Holger Prokisch, Christian Herder, Barbara Thorand, Harald Grallert

**Affiliations:** Institute of Epidemiology II, Helmholtz Zentrum München, German Research Center for Environmental Health, Neuherberg, Germany; Research Unit of Molecular Epidemiology, Helmholtz Zentrum München, German Research Center for Environmental Health, Neuherberg, Germany; German Center for Diabetes Research (DZD e.V.), Neuherberg, Germany; Institute of Computational Biology, Helmholtz Zentrum München, German Research Center for Environmental Health, Neuherberg, Germany; Interfaculty Institute for Genetics and Functional Genomics, University Medicine and Ernst Moritz Arndt University Greifswald, Greifswald, Germany; Institute of Human Genetics, Helmholtz Zentrum München, German Research Center for Environmental Health, Neuherberg, Germany; Institute of Human Genetics, Technical University Munich, Munich, Germany; Institute for Clinical Diabetology, German Diabetes Center, Leibniz Center for Diabetes Research at Heinrich Heine University Düsseldorf, Düsseldorf, Germany; German Center for Diabetes Research (DZD e.V.), Partner Site Düsseldorf, Germany; Institute of Biometrics and Epidemiology, German Diabetes Center, Leibniz Center for Diabetes Research at Heinrich Heine University Düsseldorf, Düsseldorf, Germany; Department of Endocrinology and Diabetology, University Hospital Düsseldorf, Düsseldorf, Germany; Computational Medicine, Institute of Health Sciences, University of Oulu, Oulu, Finland; NMR Metabolomics Laboratory, School of Pharmacy, University of Eastern Finland, Kuopio, Finland; Oulu University Hospital, Oulu, Finland; Computational Medicine, School of Social and Community Medicine and the Medical Research Council Integrative Epidemiology Unit, University of Bristol, Bristol, UK; Department of Nutrition and Food Sciences, Nutritional Epidemiology, University of Bonn, Bonn, Germany; Department of Epidemiology, German Institute of Human Nutrition Potsdam-Rehbrücke, Nuthetal, Germany; Department of Mathematics, Technische Universität München, Garching, Germany; Institute of Bioinformatics and Systems Biology, Helmholtz Zentrum München, German Research Center for Environmental Health, Neuherberg, Germany; Department of Physiology and Biophysics, Weill Cornell Medical College in Qatar, Education City, Qatar Foundation, Doha, Qatar; Hannover Unified Biobank, Hannover Medical School, Hannover, Germany; Deutsches Forschungszentrum für Herz-Kreislauferkrankungen, Munich Heart Alliance, Munich, Germany

**Keywords:** Metabolomics, Transcriptomics, Weight change, Obesity, Molecular epidemiology, Bioinformatics

## Abstract

**Background:**

Excess body weight is a major risk factor for cardiometabolic diseases. The complex molecular mechanisms of body weight change-induced metabolic perturbations are not fully understood. Specifically, in-depth molecular characterization of long-term body weight change in the general population is lacking. Here, we pursued a multi-omic approach to comprehensively study metabolic consequences of body weight change during a seven-year follow-up in a large prospective study.

**Methods:**

We used data from the population-based Cooperative Health Research in the Region of Augsburg (KORA) S4/F4 cohort. At follow-up (F4), two-platform serum metabolomics and whole blood gene expression measurements were obtained for 1,631 and 689 participants, respectively. Using weighted correlation network analysis, omics data were clustered into modules of closely connected molecules, followed by the formation of a partial correlation network from the modules. Association of the omics modules with previous annual percentage weight change was then determined using linear models. In addition, we performed pathway enrichment analyses, stability analyses, and assessed the relation of the omics modules with clinical traits.

**Results:**

Four metabolite and two gene expression modules were significantly and stably associated with body weight change (*P-*values ranging from 1.9 × 10^−4^ to 1.2 × 10^−24^). The four metabolite modules covered major branches of metabolism, with VLDL, LDL and large HDL subclasses, triglycerides, branched-chain amino acids and markers of energy metabolism among the main representative molecules. One gene expression module suggests a role of weight change in red blood cell development. The other gene expression module largely overlaps with the lipid-leukocyte (LL) module previously reported to interact with serum metabolites, for which we identify additional co-expressed genes. The omics modules were interrelated and showed cross-sectional associations with clinical traits. Moreover, weight gain and weight loss showed largely opposing associations with the omics modules.

**Conclusions:**

Long-term weight change in the general population globally associates with serum metabolite concentrations. An integrated metabolomics and transcriptomics approach improved the understanding of molecular mechanisms underlying the association of weight gain with changes in lipid and amino acid metabolism, insulin sensitivity, mitochondrial function as well as blood cell development and function.

**Electronic supplementary material:**

The online version of this article (doi:10.1186/s12916-015-0282-y) contains supplementary material, which is available to authorized users.

## Background

With an estimated 671 million obese individuals worldwide in 2013 [1], obesity has reached epidemic proportions. Considering the manifold health problems associated with excess body weight, including cardiovascular disease and type 2 diabetes, obesity poses a serious public health problem [2]. Understanding the mechanisms by which excess body weight contributes to cardiometabolic risk is a prerequisite for advances in therapeutic approaches. Despite extensive research, however, the complex molecular basis of body weight-related metabolic perturbations is not fully understood.

Advances in the field of high-throughput omics technologies, including metabolomics and transcriptomics, offer the opportunity to simultaneously measure hundreds or thousands of molecules, for example, metabolites and gene transcripts, thereby allowing a deeper characterization of obesity-related pathomechanisms on a molecular level [3]. In recent years, a number of cross-sectional efforts suggested a relationship between obesity and the human blood metabolome (for example, [4-6]) and transcriptome (for example, [7,8]), which extend to different tissues such as adipose tissue [9]. In addition, weight loss upon behavioral intervention was associated with changes in the blood metabolome [5,10], suggesting that the observed obesity-related molecular signatures are at least in part reversible.

However, the effect of long-term body weight change on the human blood metabolome and transcriptome in the general population – rather than under clinical settings – is less well explored. Few prospective studies have investigated the association of body weight change with concentrations of a larger set of metabolites in healthy subjects and these are restricted to a panel of lipoprotein subclasses [11,12]. In addition, although multi-omic approaches have been fruitful in different applications to enhance the understanding of complex molecular pathways (for example, [13-15]), the potential of integrating multiple omics techniques has rarely been used in the study of weight change-associated metabolic effects in humans [5].

Here, we used data from Cooperative Health Research in the Region of Augsburg (KORA) S4/F4, which constitutes a large phenotypically and molecularly well-characterized population-based cohort. We aimed to characterize associations of body weight change over a seven-year follow-up period with serum metabolomics and whole blood transcriptomics data assessed at follow-up, to determine distinct groups of molecules associated with weight change using weighted correlation network analysis (WGCNA), to study the interrelation of these groups using partial correlation networks, to investigate the stability of the findings in relevant subgroups and upon additional multivariable adjustment, for example, of subjects with weight gain versus weight reduction, and to determine the relation of the identified omics signatures with clinical traits.

## Methods

### Ethics statement

Written informed consent was obtained from all participants. The KORA studies have been approved by the ethics committee of the Bavarian Medical Association.

### Study population

KORA (Cooperative Health Research in the Augsburg Region) is a research platform of independent population-based health surveys and subsequent follow-up examinations of community-dwelling adults living in the region of Augsburg in Southern Germany. Study design, sampling method and data collection have been described in detail elsewhere [16]. The KORA S4 survey (1999 to 2001) comprised 4,261 participants, 25 to 74 years old [17]. Of these, 3,080 subjects participated in the follow-up examination KORA F4 (2006 to 2008). The present study is based on a subsample of 1,658 participants of KORA S4/F4 with metabolomics data from two platforms available. Gene expression data were available for a subsample of 703 subjects, 62 to 77 years old in F4. Women who were pregnant at the time of the examinations or during the follow-up period were excluded prior to analysis.

### Anthropometric measurements and interviews

In both examinations, body weight, height, waist and hip circumference as well as systolic and diastolic blood pressure were measured using standard protocols as described elsewhere [18]. Information on lifestyle factors and comorbidities was collected in a structured interview by trained interviewers. Intake of medication within seven days prior to examination was recorded with the IDOM-Software [19]. We combined information obtained in KORA S4 and F4 to determine changes in the variables during the follow-up period. Change in lifestyle factors, comorbidities and medication was defined as described in Additional file 1.

### Laboratory analyses

At the follow-up examination, blood samples were collected during study center visits between 8 a.m. and 11 a.m., after subjects were instructed to fast overnight for at least eight hours. Whole blood was collected using PAXgene Blood RNA tubes (BD, Heidelberg, Germany) and stored at −80°C until analysis. Red blood cell (RBC) count, hematocrit, mean corpuscular hemoglobin (MCH), mean corpuscular haemoglobin concentration (MCHC) as well as mean cell volume of erythrocytes (MCV) were measured with the impedance and the cyanmethemoglobin method using the LH 750 Hematology Analyzer (Beckman Coulter, Brea, CA, USA). Glycated hemoglobin (HbA1c) was determined using the high performance liquid chromatography method (HA 8160, Menarini, Florence, Italy). For serum collection, blood was drawn into serum gel S-Monovette tubes (Sarstedt, Nümbrecht, Germany), gently inverted two to three times and rested for 30 minutes at room temperature to obtain complete coagulation. The material was then centrifuged for 10 minutes (2,750 g at 15°C). Serum was aliquoted into synthetic straws which were kept for a maximum of six hours at 4°C before storage at −80°C until analysis. Fasting glucose levels (FGlc) and glucose levels two hours post challenge during an oral glucose tolerance test (2hGlc) were assessed with the hexokinase method (GLU Flex), low (LDL) and high density lipoprotein (HDL) cholesterol levels with the CHOD-PAP method (LDL: ALDL Flex, HDL: AHDL Flex), and fasting triglyceride (TG) levels with the GPO-PAP method (TGL Flex; all assays from Dade Behring, Eschborn, Germany). C-reactive protein was measured with nephelometry on a BN II using reagents from Siemens (Eschborn, Germany).

### Metabolomics analyses

Metabolite detection and quantification were performed on two different platforms, a commercial mass spectrometry (MS)-based platform at the company Metabolon Inc. (Durham, NC, USA) (*N* = 1,768) [20,21] and a nuclear magnetic resonance (NMR) spectroscopy-based platform (*N* = 1,788) [22].

#### Metabolon platform

The analytical platform developed by Metabolon is based on two ultrahigh-performance liquid chromatography/tandem mass spectrometry (UHPLC/MS/MS2) injections and one gas chromatography/mass spectrometry (GC/MS) injection per sample. The two UHPLC injections were optimized for basic and acidic species, respectively. More detail is given in [20] and [21]. The platform provides relative quantification for a total of 517 compounds, 325 of which could be identified based on a standard library of MS/MS spectra. The identified small molecules cover a large number of metabolite classes, including fatty acids, ketone bodies, glycerophospholipids, sphingolipids, acylcarnitines, bile acid metabolites, amino acids, peptides, carbohydrates, xenobiotics, vitamins and nucleotide metabolites. The full list of metabolites with pathway annotations is given in Table S1 in Additional file 2.

Technical effects were controlled for by dividing the metabolite concentration values by the median value of samples measured on the same day for each metabolite. See Figure S1 in Additional file 3 for the distribution of the variability (expressed as coefficient of variation (CV)) of the day medians across the metabolites. Metabolite medians varied with a median CV of 0.494 (range 0.307 to 1.220). In addition, outlier values of >4 standard deviations from the mean of the respective metabolite on the log_10_ scale were set to missing. Finally, 81 metabolites (42 identified, 39 unidentified) with more than 50% missing values, and 5 observations with more than 20% missings were excluded, leaving a total of 1,763 observations of 434 metabolites (281 identified, 153 unidentified) for analysis.

#### NMR spectroscopy platform

As another technique to obtain quantitative information on metabolic compounds, an NMR spectroscopy platform was used. The precise experimental methodology has been described elsewhere [13,22]. Computational strategies of metabolite identification and quantification from the NMR spectra are described by Inouye *et al.* [13]. A total of 130 metabolite concentrations and derived measures were obtained from the NMR spectroscopy platform (see Table S1 in Additional file 2 for a list of metabolites including full names and pathway annotations).

Preprocessing of NMR data was similar to Metabolon data in terms of outlier exclusion and detection rate thresholds. None of the metabolite traits had more than 50% missing values; however, four observations with more than 20% missings were excluded from the data set leaving a total of 1,784 observations for analysis.

#### Combined analysis of multiple metabolomics platforms

For 1,658 subjects, both Metabolon and NMR data were available (see Additional file 1: Table S2 in Additional file 2 and Figure S2 in Additional file 3 for information on the concordance of measurements for metabolites measured on both platforms). [M] and [N] is added to the metabolite names in this work to indicate measurement on the Metabolon and the NMR spectroscopy platform, respectively. Metabolites of both platforms were assigned to super-pathways and sub-pathways in accordance with the pathways proposed by Metabolon on the basis of Kyoto Encyclopedia of Genes and Genomes (KEGG) pathways. Thereby, super-pathways largely represent chemical classes analogous to the top-level pathway definitions in KEGG, whereas sub-pathways try to classify the metabolites according to their role in metabolism. As an example, the metabolites acetoacetate and 3-hydroxybutyrate (BHBA) were assigned to the super-pathway ‘Lipids’ and to the sub-pathway ‘Ketone bodies’, according to their role in ketogenesis.

### Gene expression measurement

RNA isolation and gene expression measurement have been described in detail elsewhere [23,24]. Gene expression data were exported to the statistical environment R, version 2.14.2 [25]. Data were quantile normalized using the R package *lumi*, version 2.8.0, from the Bioconductor platform [26].

### Statistical analysis

**Figure 1:**
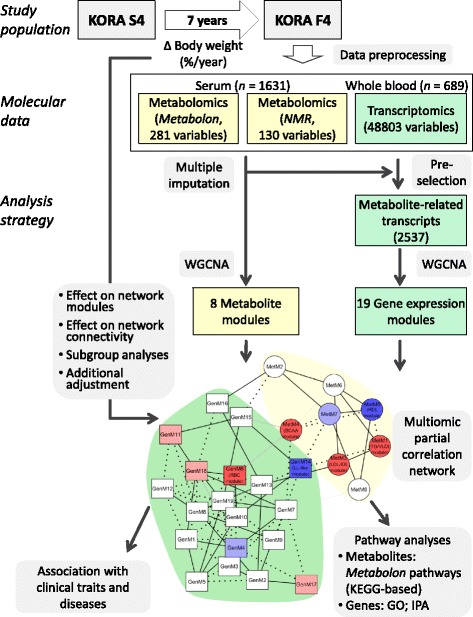
**Study design and analysis strategy.** In the network, nodes represent omics modules (circle, metabolite module (MetM); rectangle, gene expression module (GenM)), colored according to their association with annual percentage body weight change (ΔBW; red, positive association; blue, negative association; bright color, significant, *P* <1.9 × 10^–4^; light color, *P* <0.05). Edges represent partial correlations (ζ) between pairs of modules (represented by their module eigengenes), conditional on all other presented modules and the covariates age, sex, and ΔBW (solid black line, ζ >0.1; dotted black line, ζ < −0.1; solid grey line, 0.05 < ζ <0.1; dotted grey line, −0.1 < ζ < −0.05). Background color of network and boxes reflects metabolite (yellow) versus gene expression (green). GO, gene ontology; IPA, Ingenuity pathway analysis; KEGG, Kyoto Encyclopedia of Genes and Genomes; NMR, nuclear magnetic resonance; WGCNA, weighted correlation network analysis.

Of the 1,658 subjects with metabolomics data available, 22 were excluded who had a fasting duration of less than 8 hours, and 5 were excluded who had outlying values in body weight change, defined as values outside mean ± 5 standard deviations, leaving 1,631 subjects (689 subjects 62 to 77 years old with gene expression data) for analysis. The overall analysis strategy is visualized in Figure 1.

#### Multiple imputation of missing values

Data from both metabolomics platforms contained a large number of missing values. These were treated with multiple imputation by chained equations (MICE) using the R package *mice*, version 2.21 [27]. Few missing values in phenotypic variables were imputed in the same imputation process. Twenty imputed data sets were generated with 10 iterations each. See Additional file 1: Table S3 in Additional file 2, and Figures S3 to S7 in Additional file 3 for descriptives of missing values in the data set, for considerations on missing value handling, for the precise imputation settings and for imputation diagnostics.

#### Weighted correlation network analysis (WGCNA)

Previous studies have shown that clusters of related genes may be more reproducibly associated with a phenotype or disease than single genes [28] and that testing groups of metabolites instead of single metabolites improved power in a genome-wide association study [29]. Furthermore, high correlations were observed within groups of molecules in the metabolomics and transcriptomics data sets in this study. Thus, our strategy was – in addition to providing single metabolite/transcript associations – to cluster metabolomics and transcriptomics data prior to testing the association with body weight change, in order to obtain joint association signals of groups of correlated metabolites/transcripts. Specifically, metabolite and transcript levels determined at follow-up were clustered by means of WGCNA using the R package *WGCNA*, version 1.34 [30,31].

Transcripts were pre-selected based on their association with metabolites to reduce the number of transcripts to those related to the blood metabolome, aiming to improve the power and specificity to observe ΔBW-related transcripts that are relevant with regard to metabolic processes. Prior to analysis, gene expression data were log_2_ transformed and adjusted for microarray-specific technical variables (RNA integrity number, amplification plate indicators as well as sample storage time) [24] by determining the residuals from linear models of gene expression on these variables. Then, association with metabolites was determined using linear models with transformed metabolite as response and adjusted transcript as covariate, adjusting for age and sex, and a linear model additionally adjusted for body weight and ΔBW to avoid the selection of transcripts related to metabolites due to their common association with these variables. A total of 2,537 transcripts with at least a suggestive association (*P* <10^−5^) in both models were selected.

WGCNA was jointly applied to the 411 serum metabolites and derived measures (281 identified metabolites from the Metabolon platform and 130 metabolites and derived measures from the NMR spectroscopy platform, see Table S1 in Additional file 2; *N* = 1,631) and separately to the 2,537 metabolite-related transcripts (*N* = 689). Briefly, the topological overlap matrix (TOM) was derived from the Pearson’s correlations among the variables using the signed topological overlap dissimilarity measure [30]. Thereby, a soft-thresholding power of 13 (metabolite network) and 8 (transcript network) was selected based on the scale-free topology criterion (Figure S8 in Additional file 3). The TOM was then subjected to hierarchical clustering with distance between clusters defined through average linkage, and distinct clusters (‘modules’) were derived using a dynamic tree cutting algorithm with a minimum module size of five molecules [32], followed by merging closely correlated modules at a dendrogram height of 0.25 (see Figure S9 in Additional file 3 for the resulting cluster dendrograms). For each module, the module eigengene (ME), a module-representative variable, also interpretable as the ‘center’ of the module, was derived as the first principal component of a principal component analysis on the scaled matrix of molecules corresponding to the respective module. Strength of module membership, that is, the contribution of the molecules to the module, was computed as the correlation of each molecule with the respective ME.

#### Modeling association with body weight change

Linear models were used to determine the association of body weight change with the metabolite and gene expression modules. Thereby, body weight change was defined as annual percentage body weight change: ΔBW = 100% * ((weight (F4) - weight (S4))/weight (S4))/follow-up years, where weight gain was coded as positive weight change, and weight loss as negative weight change. We decided to study ΔBW rather than change in body mass index (ΔBMI), since in adults, ΔBW might be considered a more appropriate measure of weight change. When looking at change over time in adults, the division by squared height (as BMI = BW/(body height)^2^) does not improve accuracy but might rather add noise due to measurement error of height measurement, and causes an unwanted increase in BMI due to height shrinkage during aging. However, ΔBW and ΔBMI correlate highly (correlation coefficient 0.98), so that results should not be very different.

Each ME was modeled as response variable, and ΔBW, sex, age and body weight at baseline were modeled as covariates. The effects of ΔBW on MEs were tested using Wald tests, and *P*-values were corrected for multiple testing using the Bonferroni method (at *P* <0.05/(number of modules)). Association of single metabolites and metabolite-related transcripts with ΔBW were assessed in a similar way, using significance levels of *P* <0.05/411 and *P* <0.05/2,537, respectively.

The chosen modeling strategy is restricted to finding linear associations between ΔBW and MEs/molecules across the complete ΔBW range, comprising both subjects with weight loss (negative ΔBW) and weight gain (positive ΔBW). Assuming that weight loss and weight gain might not show strictly opposing metabolic effects, the analysis was repeated stratified to the groups with weight loss and weight gain. Formally, the above mentioned model was extended by subgroup index as covariate and by interaction terms of the subgroup index with ΔBW and the other covariates. Similarly, subgroup analyses were performed in obese (*N* = 426; defined as BMI >30) and non-obese (*N* = 1,205) subjects, in central obese (*N* = 522; defined as waist-hip ratio >1 in men and waist-hip ratio >0.85 in women) and not central obese (*N* = 1,109) subjects, in men (*N* = 828) and women (*N* = 803), as well as in subjects >55 years old (*N* = 855) and >55 years old (*N* = 776) at baseline.

Furthermore, as an explorative approach towards a biological explanation of the observed associations, we studied their sensitivity through additional adjustment for three groups of variables. The first model was adjusted for changes in lifestyle factors during follow-up, including change in physical activity, smoking, alcohol consumption and sleeping behavior as well as nutrition habits at baseline. The second model was adjusted for incident comorbidities during follow-up, including diabetes, cancer, myocardial infarction and stroke. The third model was adjusted for changes in medication, including beta blocker, metformin, other anti-diabetic medication, systemic corticosteroids, oral contraceptives and antidepressants. [See Additional file 1 for definitions of these variables].

Finally, cross-sectional associations of modules with binary and log-transformed continuous clinical traits in KORA F4 (metabolic syndrome, myocardial infarction (MI), stroke, HDL cholesterol, LDL cholesterol, FGlc, 2hGlc, HbA1c, systolic and diastolic blood pressure, C-reactive protein) were investigated by means of logistic and linear regression models, respectively, adjusted for age, sex, body weight, lipid-lowering medication (overall, statin, fibrates), antihypertensive medication, antidiabetic medication as well as systemic corticoid intake.

#### Pathway enrichment analyses

To formally investigate whether the identified metabolite modules were enriched for specific biological pathways (super- and sub-pathways as described above, Table S1 in Additional file 2), weighted enrichment analyses were performed as applied before in different contexts [33,34]. Briefly, for each pathway c and module m, the enrichment statistics S_cm_ was defined as the sum of module membership measures across all metabolites assigned to the respective pathway, whereas metabolites from other modules were assigned zero weight. Pathway assignment of all metabolites was randomly permuted 100,000 times, and enrichment statistics S_cm_^(perm)^ were computed. Permutation *P*-values were then defined as the number of enrichment statistics S_cm_^(perm)^ larger than the original S_cm_.

For the gene expression modules, we explored enrichment for gene ontology (GO) terms using the R packages *GO.db*, version 2.9.0, *AnnotationDbi*, version 1.22.6, and *org.Hs.eg.db*, version 2.9.0. Furthermore, the commercial software Ingenuity Pathway Analysis (IPA) was applied to identify enriched canonical pathways (IPA build version 312825 M, content version 18841524, release date: 24 June 2014; analysis date: 4 July 2014 [35]). The reference set was restricted to genes represented on the IlluminaHT-12 v3 BeadChip, and only human annotations were considered. In case multiple probes mapped to one gene, the probe exhibiting the largest module membership was considered for downstream analyses. Pathway analyses were performed with IPA’s Core Analysis module. Both GO and IPA enrichment analyses are based on Fisher’s exact test.

#### Construction of a multi-omics network

A partial correlation network was constructed from the modules significantly associated with ΔBW as described in detail [36]. For each pair of modules, the partial correlation coefficient of the respective MEs was calculated as the Pearson’s correlation coefficient of the residuals of the two MEs with regard to all other MEs, as well as sex, age, body weight and ΔBW. Since strong interrelationships among the modules might result in spurious negative partial correlations, pairwise marginal correlation (that is, the Pearson’s correlation, uncorrected for any other variables) was taken as a prerequisite.

Effects on inter- and intra-module connectivity within the multi-omics network were studied as follows: inter- and intra-module connectivity was defined as the correlation of the MEs between two modules, and the average module membership strength (definition see above) across the module members of a certain module, respectively. Significance of the difference in inter-/intra-module connectivity between the groups of weight gain and weight loss was determined through permutation testing (where weight change status was randomly shuffled).

All statistical analyses were performed in R, version 3.0.1 [25].

## Results and discussion

Using data from the population-based KORA S4/F4 cohort, we characterized the multi-omic signature associated with body weight change over a seven-year follow-up period. Two-platform serum metabolomics and whole blood transcriptomics measurements were available from the follow-up examination F4 for 1,631 and 689 participants, respectively (Table 1). Clustering of the 411 metabolites and the 2,537 metabolite-related transcripts generated eight metabolite modules (MetM) and 19 gene expression modules (GenM), respectively (Figure 1).

**Table 1:** **Characteristics of the study population**

**Variable**	**Metabolomics data (** ***N*** **= 1,631)**	**Combined metabolomics and transcriptomics data (** ***N*** **= 689)**
	**Mean (sd)**	**Mean (sd)**
Body weight (kg), baseline	78.3 (14.7)	78.5 (13.2)
Body weight (kg), follow-up	79.7 (15.6)	79.3 (13.8)
Δ Body weight (%)	1.8 (6.8)	1.0 (6.5)
Δ Body weight/year (%)	0.3 (1.0)	0.1 (0.9)
BMI (kg/m^2^), baseline	27.7 (4.5)	28.5 (4.3)
BMI (kg/m^2^), follow-up	28.2 (4.7)	28.8 (4.5)
Age (years), baseline	54.2 (8.7)	61.8 (4.3)
Age (years), follow-up	61.2 (8.7)	68.8 (4.3)
	**Relative frequency (%)**	**Relative frequency (%)**
Sex (male/female)	50.8/49.2	50.1/49.9
Weight change direction (reduction/gain)	39.3/60.7	45.9/54.1

### Body weight change is globally associated with the metabolite profile

Four of the eight MetMs were significantly associated with annual percentage body weight change (ΔBW), in linear models adjusted for age, sex and baseline body weight (positive associations for MetM1, *P =* 1.2 × 10^−24^, MetM3, *P =* 2.2 × 10^−4^, and MetM4, *P =* 7.3 × 10^−17^; negative association for MetM5, *P =* 1.7 × 10^−14^; all significant after Bonferroni correction for 27 modules). These four modules comprised a total of 147 metabolites. Together, these metabolites covered major branches of metabolism captured by the metabolomics platforms, including lipid metabolism, amino acids and peptides, carbohydrate metabolism, cofactors and vitamins, and energy metabolism (Figure S10 in Additional file 3). This suggests a global association of body weight change with the serum metabolome.

**Figure 2:**
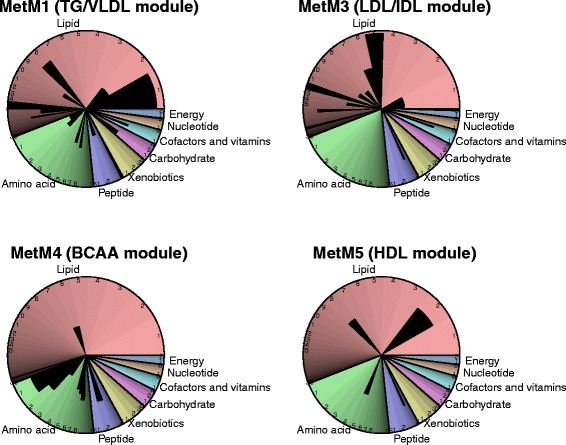
**Coverage of the serum metabolome by the metabolite modules (MetM) related to body weight change (ΔBW).** Pie chart with color indicating super-/sub-pathways (see legend of Figure S10 in Additional file 3), and size of segments representing the number of metabolites in the data set corresponding to the respective sub-pathway. Sorted by pathway size. Black wedges represent the number of metabolites from the respective module significantly associated with ΔBW in the respective sub-pathway. For instance, metabolites from lipid sub-pathway 2 (‘HDL’) contributed most strongly to MetM5 (the ‘HDL module), in line with a significant enrichment (see text). BCAA, branched chain amino acids; HDL, high density lipoprotein; IDL, intermediate density lipoprotein; LDL, low density lipoprotein; TG, triglycerides; VLDL, very low density lipoprotein.

**Figure 3:**
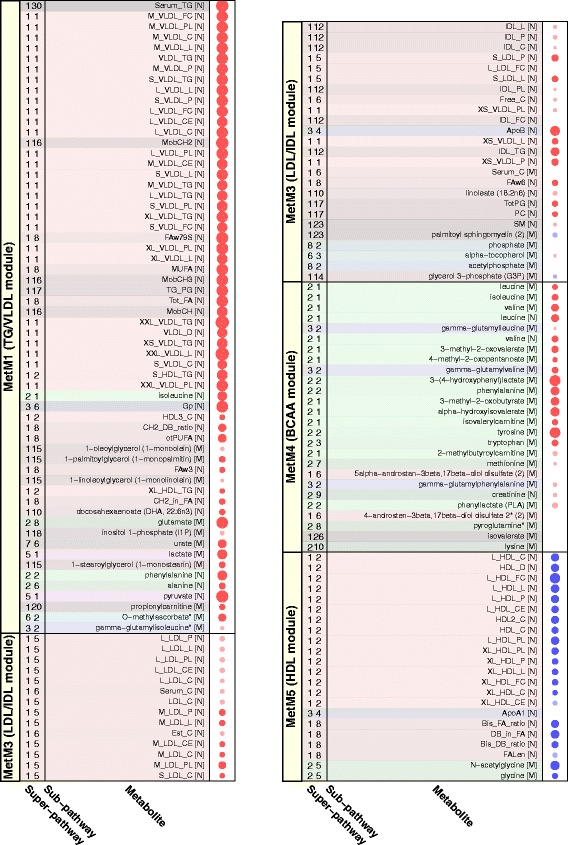
**Association of body weight change (ΔBW) with members of associated metabolite modules (MetM).** Bubbles represent effect strengths and significance, see legend of Figure 6. Models were adjusted for age, sex and baseline body weight. For single metabolites, the significance threshold was chosen as *P* <1.2 × 10^−4^ corresponding to Bonferroni correction for 411 tests. Background colors correspond to super- and sub-pathway annotations, see legend of Figure S10 in Additional file 3. Note that all effects are shown per unit of ΔBW, which is a variable spanning the whole weight change range (with weight loss coded as negative ΔBW values and weight gain as positive ΔBW values). Thus, effects have to be interpreted as the average linear association across the weight change range, and effect directions have to be inverted to construe the association with weight reduction. Using the example of Serum_TG [N], the positive association of ΔBW with Serum_TG [N] can be interpreted as increase in serum triglyceride (TG) levels with increasing weight gain, and as decrease in serum TG levels with increasing weight loss.

MetM1 (comprising 60 metabolites) was strongly determined by constituents of all very low density lipoprotein (VLDL) subclasses, total serum TGs, TGs in small HDL (S-HDL) and measures of primarily saturated and monounsaturated fatty acids (Figure 2), which all showed a module membership strength (that is, correlation with the module ‘center’) of above 0.8 (Figure 3, Table S4 in Additional file 2). Together with isoleucine [N], glycoprotein (Gp [N]), glutamate [M], ureate [M], lactate [M], phenylalanine [N] and pyruvate [N], these most connected metabolites were also most strongly associated with ΔBW in the single metabolite models (Figure 3). When a formal enrichment analysis was performed, MetM1 was significantly enriched for metabolites belonging to the super-pathway ‘Lipids’ and the sub-pathways ‘VLDL’ and ‘Triacylglycerol’ (all *P <* 10^−5^), confirming the predominant role of these metabolic pathways for MetM1. MetM3 (comprising 39 metabolites) was mainly driven by constituents of LDL and intermediate density lipoprotein (IDL) subclasses and very small VLDL (XS-VLDL), measures of serum cholesterol as well as apolipoprotein B (module membership strengths >0.8, Figures 2 and 3, Table S4 in Additional file 2). In addition, a significant enrichment for the super-pathway ‘Lipids’ and the sub-pathways ‘LDL’ and ‘IDL’ was observed (*P* <10^−5^). The most contributing metabolites of MetM4 (comprising 26 metabolites) were the branched chain amino acids (BCAAs) valine, leucine and isoleucine, and the peptide gamma-glutamylleucine, with a significant enrichment for the super-pathway ‘Amino acids’ and the sub-pathway ‘Valine, leucine and isoleucine metabolism’ (*P* <10^−5^). Finally, MetM5 comprised 22 metabolites and was mostly driven by constituents of large (L-) and very large (XL-) HDL as well as apolipoprotein A1, with a significant enrichment for the super-pathway ‘Lipids’ (*P =* 1.6 × 10^−4^) and the sub-pathway ‘HDL’ (*P* <10^−5^). To improve readability, the four ΔBW-associated metabolite modules are hereafter referred to as ‘TG/VLDL module’ (MetM1), ‘LDL/IDL module’ (MetM3), ‘BCAA module’ (MetM4) and ‘HDL module’ (MetM5), according to their significantly enriched sub-pathways.

These results demonstrate that ΔBW strongly associates with lipoprotein constituents (see also Figure S11 in Additional file 3), amino acids and peptides, as well as metabolites of energy metabolism, and that clustering helped to reveal pathways jointly and strongly associated with ΔBW.

### The metabolic signature associated with body weight change is consistent with known pathophysiology of obesity

Overall, the metabolite signature associated with ΔBW concurred with known aspects of the pathophysiology of weight change and obesity. The associations of ΔBW with lipoprotein subclasses (positive association with VLDL, LDL and S-HDL subclasses, negative association with larger HDL particles and with HDL and LDL particle size; Figure 3 and Figure S11 in Additional file 3) are in agreement with the observations of two smaller prospective studies that analyzed the effect of weight change over similar time periods (9 and 6.5 years, respectively) on lipoprotein subclasses [11,12]. Specifically, ΔBW was positively associated with increases in VLDL and LDL subclasses, and with decreases in L-HDL, whereas S-HDL behaved oppositely [12]. ΔBW was also negatively related to LDL and HDL particle sizes [11,12]. The clustering of S_HDL_TG [N] (TG in S-HDL) within the TG/VLDL module in our study was also in agreement with its close correlation with VLDL subclasses in Inouye *et al.* [13], where S-HDL behaved differently from larger HDL subclasses with regard to metabolite-transcript associations.

Mechanisms by which body weight increase gives rise to the described changes may include an increased release of free fatty acids from adipose tissue, triggering hepatic TG and VLDL production [37] and increasing the activity of hepatic lipase [38]. Hepatic lipase is involved in the exchange of TGs from VLDL against cholesterol esters from HDL, thereby promoting the production of small dense LDL. Together with phospholipid transfer protein (PLTP) and cholesterol ester transfer protein (CETP), which also show increased levels upon obesity [39], hepatic lipase is centrally involved in regulating HDL particle size. Interestingly, this was reflected in oppositional associations of genetic variants in the respective genes *LIPC*, *PLTP* and *CETP* with small versus large HDL subclasses [40].

**Figure 4:**
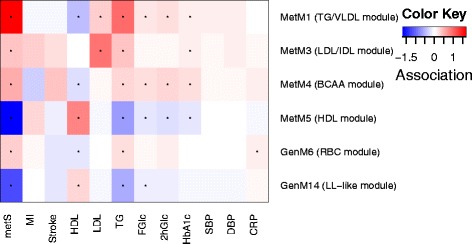
**Association of the identified omics modules with clinical traits.** Results are derived from linear (continuous traits, log-transformed) and logistic (binary traits) regression models adjusted for age, sex, body weight, lipid-lowering medication (overall, statin, fibrates), antihypertensive medication, antidiabetic medication as well as systemic corticoid intake. Significant associations (*P* <6.9 × 10^−4^ corresponding to Bonferroni correction for 72 tests) are denoted as black stars. 2hGlc, glucose two hours post challenge during an oral glucose tolerance test; CRP, C-reactive protein; DBP, diastolic blood pressure; FGlc, fasting glucose; GenM, gene expression module; HDL, high density lipoprotein cholesterol; LDL, low density lipoprotein cholesterol; MetM, metabolite module; metS, metabolic syndrome; MI, myocardial infarction; SBP, systolic blood pressure; TG, trigylcerides. See Table S5 in Additional file 2 for descriptives of clinical traits. For disease definitions, see Methods.

The lipoprotein signature related to positive ΔBW (that is, weight gain) largely corresponds to an unfavorable, atherogenic lipid profile. For instance, large VLDL and small HDL particles were found to be positively, and larger HDL particles to be negatively, associated with coronary artery disease severity [41]. In a large prospective cohort of 4,594 initially healthy adults, a lipoprotein pattern characterized by decreased L-HDL, increased S-/M-LDL, and increased TGs was associated with an increased cardiovascular disease incidence after a mean follow-up of 12 years [42]. Furthermore, VLDL particle size, which was positively associated with ΔBW in our study, predicted type 2 diabetes incidence over a 13-year follow-up of 26,836 initially healthy women [43]. In line with these findings, we observed a strong positive association of the TG/VLDL module and a strong negative association of the HDL module with markers of insulin resistance (HbA1c: *P =* 1.9 × 10^−5^ and 7.0 × 10^−7^; 2hGlc: *P =* 2.1 × 10^−9^ and 5.1 × 10^−8^, respectively) determined at follow-up (Figure 4, Table S5).

ΔBW was strongly associated with amino acid concentrations, most prominently BCAAs, phenylalanine, tyrosine and glutamate. The increase of these amino acids in obesity has long been known [44], and has also been observed in more recent studies (for example, [4]). The underlying mechanism might be an impaired catabolism of BCAA upon obesity [4]. Experimental studies show that BCAAs inhibit the insulin receptor substrate via the mTOR/p70S6K/S6K pathway [45]. Accordingly, in the study by Newgard *et al.* [4], addition of BCAAs to a high-fat diet in rats promoted the development of insulin resistance. Recently, BCAAs, phenylalanine and tyrosine were shown to associate with future insulin resistance [46], future type 2 diabetes [47] and prevalent metabolic syndrome [48]. In our study, the BCAA module associated positively with markers of insulin resistance (HbA1c: *P* = 7.2 × 10^−10^; 2hGlc: *P* = 9.0 × 10^−9^) and metabolic syndrome prevalence (*P* = 3.8 × 10^−5^) (Figure 4).

### The metabolic signature associated with body weight change points towards mitochondrial dysfunction

The positive association of ΔBW with several metabolites in the TG/VLDL and BCAA modules is supportive of a link between body weight gain and mitochondrial dysfunction. Mitochondria are important organelles in the regulation of metabolism [49]. As a main function, they produce adenosine triphosphate (ATP) from carbohydrates, fats and proteins via the tricarboxylic acid cycle, which is supplied with pyruvate (from glycolysis), acetyl-CoA (from β-oxidation) and amino acid metabolites (from protein catabolism) [49,50]. In states of insufficient oxygen supply or mitochondrial dysfunction, pyruvate is not imported into the mitochondria but instead converted to lactate via lactic acid fermentation and to alanine via transamination. Obesity is associated with decreased fatty acid β-oxidation so that obese individuals are more dependent on the glycolytic pathway for ATP production [50], resulting in an increased pyruvate production. At the same time, obesity is associated with diminished mitochondrial biogenesis [49], reduced mitochondrial size and diminished respiratory chain activity [51]. These effects might explain increased circulating levels of pyruvate, lactate and alanine upon weight gain in our study. Concentrations of these metabolites have previously been shown to be elevated in obesity [4], and lactate levels increased upon weight gain during chemotherapy in early breast cancer patients [52]. Furthermore, concentrations of pyruvate, lactate and alanine were found to be predictive of future glucose intolerance, independent of body mass [46].

All three metabolites clustered in the TG/VLDL module, together with VLDL subclasses, fatty acids, propionylcarnitine and phenylalanine. The blood concentrations of fatty acids and circulating acylcarnitines (among them propionylcarnitine) were found to be increased in obese subjects [4]. Elevated levels of these metabolites are also thought to be linked to mitochondrial dysfunction [49]. In addition, aluminum-induced mitochondrial dysfunction was found to promote VLDL secretion in human hepatocytes [53], suggesting a role of mitochondrial dysfunction as a further link between ΔBW and dyslipidemia as well as cardiometabolic disease.

Furthermore, two C5 acylcarnitines, isovalerylcarnitine and 2 − methylbutyroylcarnitine, BCAAs, tyrosine, tryptophan and creatinine clustered in the BCAA module. Short-chain acylcarnitines (C4 and C5) are products of BCAA catabolism. As catabolism of BCAAs is performed in the mitochondrial matrix, elevated BCAA levels could be an indicator for mitochondrial dysfunction [49]. A recent retrospective study found primary mitochondrial respiratory chain disease to be significantly associated with elevated BCAA levels [54]. Moreover, respiratory chain inhibition in cultured muscle cells was found to be significantly associated with a reduced uptake of several other amino acids, among them phenylalanine, tyrosine and tryptophan (together with a greater secretion of lactate, alanine and creatine) [55]. Together, metabolites of the TG/VLDL and the BCAA module provide a link between mitochondrial dysfunction and body weight gain already in a non-obese state.

### Individual metabolite associations with body weight change provide further interesting insights

As a result of the minimum module size of five molecules chosen in WGCNA, metabolites reflecting biological pathways that are represented by fewer than five correlated metabolites on the metabolomics platforms are less likely to cluster in modules sharing association with ΔBW. Since these might, nevertheless, be interesting, we provide the overall single metabolite association results in Table S4 in Additional file 2. These include the positive association of ΔBW with the tryptophan metabolites hydroxytryptophan [M] and kynurenine [M], which are successors of tryptophan in the serotonin and niacin biosynthesis pathways, respectively, and negative association with serotonin (5HT) [M]. They also include the positive association with the xenobiotics caffeine [M] and piperine [M]. Increased concentrations in these substances may result either from an increased consumption of coffee or caffeinated drinks, and herbs or spices, or from a lower degradation or excretion. Furthermore, we observed a negative association of ΔBW with quinate [M] and catechol sulfate [M], a positive association with bradykinin, des-arg(9) [M], the active metabolite of the vasodilating peptide hormone bradykinin, and a positive association with the metabolites N1-methyl-3-pyridon-4-carboxamide [M] and N1-methyladenosine [M] from the nucleotide super-pathway.

### Body weight change associates with a gene expression module related to erythrocyte development and a lipid-leukocyte-like module

Two of the 19 gene expression modules (GenMs) were significantly associated with ΔBW in linear models adjusted for age, sex and baseline body weight (positive association for GenM6, *P* = 3.8 × 10^−12^; negative association for GenM14, *P* = 1.9 × 10^−4^).

**Figure 5:**
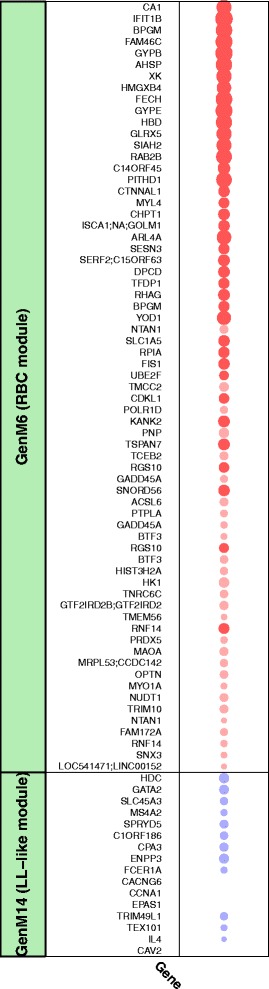
**Association of body weight change (ΔBW) with members of associated gene expression modules (GenM).** Bubbles represent effect strengths and significance, see legend of Figure 6. Models were adjusted for age, sex and baseline body weight. For single transcripts, the significance threshold was chosen as *P* <2.0 × 10^−5^ corresponding to Bonferroni correction for 2,537 metabolite-related transcripts. Genes are sorted by their module membership strength, as determined by the correlation of transcript level with the module eigengene. Gene annotations were derived from the UCSC data base. UCSC, University of California, Santa Cruz.

GenM6, which comprised 71 transcripts, showed a strong positive association with ΔBW. The core of GenM6 (module membership strength >0.8) comprised *CA1*, *IFIT1L*, *BPGM*, *FAM46C*, *GYPB*, *AHSP*, *XK*, *HMGXB4*, *FECH*, *GYPE*, *HBD* and *GLRX5* (Figure 5, Table S6 in Additional file 2). A manual literature search revealed the majority of these twelve genes as RBC-related genes, so that GenM6 was termed the ‘RBC module.’ For instance, *HBD* encodes the hemoglobin delta subunit, *AHSP* encodes α-hemoglobin stabilizing protein, *BPGM* regulates the oxygen affinity of hemoglobin, and *FECH* codes for the enzyme ferrochelatase/heme synthase which is involved in heme synthesis. A formal enrichment analysis for GO terms revealed ‘bicarbonate transport’, ‘hemoglobin metabolic process’ and ‘hemoglobin complex’ as the top three enriched biological pathways (all *P* = 8.4 × 10^−4^, not significant after multiple testing correction, Table S7 in Additional file 2). In Ingenuity pathway analysis, ‘heme biosynthesis from uroporphyrinogen-III I’ was among the top five upregulated canonical pathways (*P* = 9.9 × 10^−3^, not significant after multiple testing correction), although results are based on only one gene (*FECH*) in this pathway (Table S8 in Additional file 2).

Consequently, we hypothesized that the transcripts in the RBC module are reflective of erythrocyte development, since immature RBCs, reticulocytes, contain remnant mRNA [56] which is depleted during erythrocyte maturation. An increased hematopoiesis upon diet-induced obesity in rats has been observed, putatively through action of leptin in the bone marrow [57], whereas glycosylated hemoglobin shows an inverse relationship with erythrocyte survival [58]. A shift towards a larger proportion of immature RBCs upon weight gain would be consistent with these observations. In agreement with our findings, a small transcriptomics study of obesity reported the majority of the genes significantly upregulated in obesity to be related to reticulocytes [8].

The proportion of immature reticulocytes was shown to be elevated in cardiac disease patients [59]. In a transcriptomics study of participants from the Framingham heart study, a cluster of transcripts specific to CD71+ early erythroid cells was significantly upregulated in coronary heart disease (CHD) cases compared to controls [60]. This cluster, comprising 126 transcripts, showed a large overlap with the RBC module identified in our study, with nine of the above-mentioned core transcripts being contained in this cluster. Furthermore, Zhang *et al*. observed an increased RBC count and increased hemoglobin levels in obese but not in non-obese CHD patients as compared to healthy controls [61]. Also, the obese patients were more likely to have acute coronary syndrome, which the authors attribute to a potential role of RBCs in the development of plaque instability. Our results suggest that even in non-obese subjects, weight change is related to RBC development.

Another, smaller, gene expression module (GenM14, comprising 17 transcripts) was negatively associated with ΔBW (Figure 5, Table S6 in Additional file 2). GenM14 contained all 11 transcripts of the ‘lipid-leukocyte (LL) module’ previously described as a leukocyte gene expression module strongly related to blood lipids [62] and a large number of serum metabolites including lipoprotein subclasses, lipids, glycoproteins and amino acids [13]. We therefore termed it the ‘LL-like module.’ The core of the LL-like module (module membership strength >0.8) comprised the LL genes *HDC*, *GATA2*, *SLC45A3*, *MS4A2* and *SPRYD5*. Inouye *et al*. [62] discussed this module as being involved in basophil and mast cell-related immune response and allergy. For instance, the core gene *HDC* codes for a protein converting histidine to histamine, which is secreted by basophils and mast cells in response to IgE sensitization. Accordingly, when we applied Ingenuity pathway analysis to our data, ‘FC Epsilon RI Signaling’ (*P* = 1.1 × 10^−4^), ‘Histamine Biosynthesis’ (*P* = 9.1 × 10^−4^) and ‘Airway Inflammation in Asthma’ (*P* = 3.7 × 10^−3^) were the top three canonical pathways (Table S8 in Additional file 2). Six further transcripts, *IL4*, *TRIM49L1*, *TEX101*, *EPAS1/HIF2-α*, *CCNA1* and *CAV2*, were co-expressed with the LL module genes, although being less strongly correlated with the module center (module membership strengths ranging from 0.48 to 0.54), suggesting that they might share functionality with the LL module genes. Indeed, *IL4* codes for the cytokine interleukin 4 which has long been known to induce differentiation of naïve T cells to Th2 cells that play a role in allergen response and which is secreted by basophils as a reaction to allergens [63]. *EPAS1/HIF2-α* encodes a component of the hypoxia inducible transcription factor (HIF), which regulates responses to reduced oxygen and for which also a role in regulating inflammation [64] and energy balance [65] has been reported. Of note, the association of the LL-like module with ΔBW as well as with the TG/VLDL module was negative. Although these findings are in line with the negative association between the LL module and VLDL metabolites reported by Inouye *et al.* [13], they are contradictory to an analysis by Gonen *et al.*, in which VLDL was found to trigger the release of histamine from human basophils [66]. Furthermore, obesity is a risk factor for asthma and weight gain was found to increase the risk of developing airway hyperresponsiveness [67]. It remains to be determined how these results fit with our observation of decreased expression of genes related to basophil/mast cell level or function being associated with weight gain.

Neither of the ΔBW-related GenM’s seemed to comprise genes with a well-established relationship to lipid metabolism, as might be expected after preselecting metabolite-related transcripts. Exemplarily, we looked up the genes *LIPC*, *CETP* and *PLTP* discussed above within the context of lipoprotein metabolism, as well as *ABCG1* which has been discussed together with *CETP* as a strongly upregulated transcript in adipose tissue in response to diet-induced weight loss [68]. Whereas *ABCG1* transcripts tended to show a negative association with ΔBW (best *P* = 6.7 × 10^−5^ for transcript ILMN_2329927, which clustered in GenM1), transcripts of the other three genes were not related to either ΔBW or metabolites. These results could have been expected considering the tissue origin of these proteins. In line with these findings, the strong obesity-related changes in adipose tissue gene expression were weakly represented by blood cell transcriptomics in the study by Emilsson *et al.* [7].

### Interrelation of the omics modules and sensitivity analyses

To investigate the interrelatedness of the identified ΔBW-related metabolite and gene expression modules conditional on all other modules and the above-mentioned covariates, we constructed a partial correlation network from the MEs (Figure 1; a larger illustration is provided in Figure S12 in Additional file 3). The six ΔBW-related modules were interrelated. The strongest positive partial correlation was observed between the TG/VLDL module and the LDL/IDL module (*P* = 2.3 × 10^−54^). In addition, the TG/VLDL module showed a strong negative correlation with the HDL module (*P* = 1.8 × 10^−72^) and with the LL-like module (*P* = 6.8 × 10^−29^).

**Figure 6:**
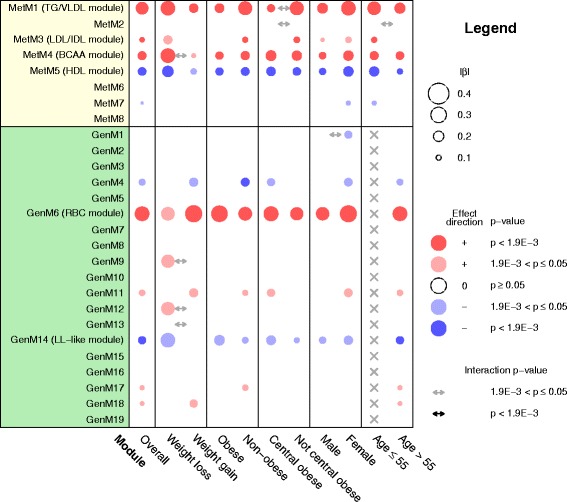
**Association of body weight change (ΔBW) with omics modules.** Shown are associations for the overall study population (column 1) and for subgroups (columns 2 to 11). Bubbles represent effect strengths and significance, as described in the legend. Models were adjusted for age, sex and baseline body weight. Significance threshold *P* <1.9 × 10^−3^ corresponds to Bonferroni correction for 27 modules. For subgroup analyses (columns 2 to 11), interaction models were fitted to obtain the main ΔBW effect in the respective subgroups, and the ΔBW:subgroup interaction effect indicating the difference in effect between the subgroups. Gene expression analysis was restricted to a subgroup of 689 subjects >55 years old at baseline, so that no effect estimates are available for the younger subgroup in this population (indicated as grey crosses). Note that all effects are shown per unit of ΔBW, which is a variable spanning the whole weight change range (with weight loss coded as negative ΔBW values and weight gain as positive ΔBW values). Thus, in the weight loss subgroup analysis, effects have to be inverted to obtain the effect per annual percentage body weight loss, and the same color of effects in the weight loss and weight gain subgroups denotes opposing effects of weight loss versus weight gain. GenM, gene expression module; MetM, metabolite module.

Next, we performed different analyses to assess the stability of the multi-omic network and its relation to ΔBW. First, we argued that metabolic effects of weight loss (negative ΔBW) and weight gain (positive ΔBW) might not be strictly opposing, and that diverging effects might remain unexplored when linear models are used. Therefore, we performed stratified analyses in the group of subjects with weight loss (*N* = 641; 316 with gene expression data) and in the group of subjects with weight gain (*N* = 990; 373 with gene expression data) (Figure 6, second and third column). Overall, weight loss and weight gain tended to show opposing associations with the modules (Figure 6: same color of circles denoting association). By trend, associations of the TG/VLDL module, the LDL/IDL module, the BCAA module, the HDL module and the LL-like module were stronger in subjects with weight loss than with weight gain. In contrast, the RBC module showed by trend a stronger association in the group with weight gain. However, none of these differences were significant (Figure 6: black arrows).

In addition, the effect of ΔBW on inter- and intra-module connectivity of the network elements was investigated, since previous studies suggested sensitivity of metabolic network topology towards external factors [13,69] (see Methods). We did not observe any significant differences in network connectivity between the groups with weight gain and weight loss (all *P* >0.01).

The generally opposing associations of weight loss and weight gain with blood metabolite concentrations are in line with the studies by Mäntyselkä *et al.* [12] and Naganuma *et al.* [11], where the majority of ΔBW associations with lipoprotein measures were linear across the weight change range, and weight loss and weight gain showed opposing effects. Interestingly, the effect of weight loss (≥5% across 6.5 years) versus stable weight on VLDL subclasses and L-HDL was stronger in absolute terms than the effect of weight gain (≥5%) versus stable weight [12]. These findings are in accordance with the stronger associations for the TG/VLDL module and the HDL module observed for weight loss in our study. Although larger studies in subjects with a larger range of weight change might have more power to differentially investigate the effects of weight loss versus weight gain, our results suggest that differences are not large and that, in general, weight loss is capable of reversing the effects of weight gain on the blood metabolome and transcriptome. Accordingly, it was shown in a randomized controlled trial that normalization of obesity led to a reversal of an unfavorable LDL subclass pattern [70].

Second, further subgroup analyses were performed, assuming that the weight change effect might depend on (central) obesity, on sex and on age (Figure 6, Figure S13 in Additional file 3). Again, no significant subgroup-specific effects were observed, although, as already mentioned above, this study might not provide sufficient power to study effect modification.

Body weight change over a period of seven years might be due to several reasons, including changes in lifestyle, the occurrence of diseases and changes in medication. For these reasons, we investigated the sensitivity of the observed associations with ΔBW towards adjustment for changes in lifestyle factors, for disease incidence and, finally, for changes in medication in three separate models (Table S9 in Additional file 2, Figure S14 in Additional file 3). None of the three models showed a change in effect sizes across the modules, indicating that the observed associations were primarily due to the change in body weight *per se* rather than the mechanisms that might have facilitated weight change. Note, however, that the majority of the variables reflecting changes in lifestyle, disease and medication were obtained from interviews and might, therefore, have insufficient accuracy. Also, nutrition was only obtained from the baseline time point so that the effect of changes could not be investigated.

Together, these results suggest that the metabolite-gene network and its relation to weight change reflect a largely stable system.

Several extensions of our study seem worthwhile. First, it would be interesting to obtain a higher resolution of body weight measurements during follow-up, as well as of metabolomics and gene expression measurements, to decipher the longitudinal sequence of metabolic changes and to study the metabolic processes related to weight cycling. An important limitation of this study is that metabolomics and transcriptomics data were not available from the baseline time point from all platforms and for all subjects. The present observational study does not allow conclusions on the effect directions underlying the observed associations of body weight change with metabolite or transcript levels. In addition, extending whole blood transcriptomics to different tissues seems extremely promising, considering that blood might only weakly reflect weight-related transcriptional changes in tissues [7] and that blood metabolites originate from different tissues. However, gene expression signatures identified in blood will be of large practical relevance since blood is most easily accessible also in a clinical setting. Also, in the context of weight change and its metabolic consequences, integrating metabolomics and blood cell transcriptomics is relevant from the perspective that blood cells may interact with blood substances in the etiology of atherosclerotic events [13].

## Conclusions

Through the integration of two-platform serum metabolomic and whole blood transcriptomic data and the formation of modules of closely connected molecules, we obtained a comprehensive characterization of the metabolomic and transcriptomic signature of body weight change over a seven-year period in a large population-based cohort. Weight gain and weight loss were strongly and opposingly associated with the blood metabolome, with VLDL, LDL and large HDL subclasses, TGs, BCAAs and markers of energy metabolism as core molecules of the four metabolite modules. These associations point towards the development of dyslipidemia, disturbed amino acid metabolism as well as mitochondrial dysfunction upon weight gain. Two weight change-related gene expression modules pinpoint reticulocytes and immune cells (mast cells, basophils) as blood cell types putatively playing a role in body weight-related blood metabolism. Metabolite and gene expression modules were associated with clinical phenotypes, suggesting a role in linking excess body weight with metabolic and cardiovascular comorbidities. Our findings also support the hypothesis that clustering omics data prior to analyzing associations with a phenotype has increased power to identify biologically relevant pathways [28,29]. The LL-like module was found to be associated with weight change, although none of the contributing genes showed a univariate association with weight change that would have passed significance after correction for multiple comparisons.

Together, our study provides evidence for a largely reversible effect of long-term body weight gain in the general population on an integrated blood metabolomic and transcriptomic network. This improves the knowledge on molecular processes elicited by weight change and potentially linking it to comorbidities.

## Additional files

Additional file 1:
**Supplementary methods.**
Additional file 2: Table S1. List of metabolites included in the analysis. **Table S2.** Cross-platform correlations of metabolites determined on the Metabolon and NMR spectroscopy platforms. **Table S3.** Metabolite transformation. **Table S4.** Association of annual percentage body weight change (ΔBW) with single metabolite concentrations. **Table S5.** Description of clinical traits in the KORA F4 study. **Table S6.** Association of annual percentage body weight change (ΔBW) with single transcript levels for the 2,537 metabolite-related transcripts. **Table S7.** Gene ontology enrichment analysis. **Table S8.** Ingenuity pathway analysis. **Table S9.** Description of lifestyle factors, disease prevalence and incidence and medication. Additional file 3: Figure S1. Distribution of the variability of the day medians across the Metabolon metabolites. **Figure S2.** Scatter plot of serum measurements on the Metabolon and NMR platforms. **Figure S3.** Missingness pattern in the metabolomics data set. **Figure S4.** Correlation among metabolites and phenotypes. **Figure S5.** Correlations of missingness with variable values. **Figure S6.** Convergence of the MICE algorithm for the metabolite with the largest proportion of missings (3-hydroxy-2-ethylpropionate [M]). **Figure S7.** Imputation diagnostics for two selected variables. **Figure S8.** Choice of the scale-free topology parameter in weighted correlation network analysis (WGCNA). **Figure S9.** Weighted correlation network analysis (WGCNA) cluster dendrograms. **Figure S10.** Joint coverage of the serum metabolome by the four metabolite modules related to annual percentage body weight change (ΔBW). **Figure S11.** Association of annual percentage body weight change (ΔBW) with lipoprotein subclasses. **Figure S12.** Multi-omic partial correlation network comprising the 8 metabolite and the 19 gene expression modules. **Figure S13.** Association of annual percentage body weight change (ΔBW) with members of associated metabolite modules (MetM) – results of the stratified analyses. **Figure S14.** Association of annual percentage body weight change (ΔBW) with omics modules adjusting for factors driving weight change.
