# Supplementary material for: Multi-omic signature of body weight change: results from a population-based cohort study
Source: BMC Med. 2015 Mar 9;13:48. doi: 10.1186/s12916-015-0282-y (PMC4367822; doi:10.1186/s12916-015-0282-y)
Supplement: Additional file 1: — Supplementary methods. [file 12916_2015_282_MOESM1_ESM.docx]

# Multi-omic signature of body weight change: results from a population-based cohort study

Simone Wahl*, Susanne Vogt*, Ferdinand Stückler, Jan Krumsiek, Jörg Bartel, Tim Kacprowski, Katharina Schramm, Maren Carstensen, Wolfgang Rathmann, Michael Roden, Carolin Jourdan, Antti J Kangas, Pasi Soininen, Mika Ala-Korpela, Ute Nöthlings, Heiner Boeing, Fabian J Theis, Christa Meisinger, Melanie Waldenberger, Karsten Suhre, Georg Homuth, Christian Gieger, Gabi Kastenmüller, Thomas Illig, Jakob Linseisen, Annette Peters, Holger Prokisch, Christian Herder, Barbara Thorand^#^, Harald Grallert^#^

Additional file 1: Supplementary methods

*,^#^ contributed equally

**Lifestyle factors, diseases and medication**

Change in lifestyle factors was determined as follows: Change in alcohol consumption was determined in g/d. Change in sleeping behaviour was categorized as “improvement”, “no change” and “worsening” of problems to fall asleep or to sleep through the night. Change in physical activity was categorized as “became active”, “no change” and “became inactive”, where subjects were categorized as “active” if they regularly spent at least one hour of moderate and vigorous physical activity per week during leisure time in summer and winter, and as “inactive”, if they spent less than one hour. Finally, change in smoking status was categorized as “started smoking”, “no change” and “quit smoking”. Nutrition habits were only assessed at baseline, using a food frequency questionnaire (based on recommendations of the German Nutrition Society). Based on how often participants reported to consume 15 different food categories, their nutrition habits were categorized as “disadvantageous”, “normal” or “advantageous”. Considering the importance of nutritional patterns on the metabolic status of the body, we decided to include this variable in the analysis, despite nutritional habits were only available from the baseline timepoint.

Change in disease was categorized as “incident disease during follow-up” and “no incident disease during follow-up”. Change in the intake of medication known to associate with weight gain, i.e. beta blocker, anti-diabetic medication without metformin, systemic corticosteroids, oral contraceptives and antidepressants [[1](#_ENREF_1), [2](#_ENREF_2)], and change in metformin intake, which is associated with weight loss [[3](#_ENREF_3)], was categorized as “started intake”, ”no change” and “stopped intake”.

The metabolic syndrome (MetS) was defined based on the Adult Treatment Panel III (ATP III) criteria [[4](#_ENREF_4)], as the presence of three out of (1) abdominal obesity (WC > 102 cm in males and > 88 cm in females), (2) high TG (≥ 150 mg/dl), (3) low HDL cholesterol (< 40 mg/dl in males and < 50 mg/dl in females), (4) hypertension (SPB ≥ 130 mmHg or DPB ≥ 85 mmHg) and (5) high fasting glucose (≥ 110 mg/dl).

**Two-platform metabolomics**

For 1658 subjects, both Metabolon and NMR data were available. Data from different metabolomics platforms have been jointly analyzed before [[5](#_ENREF_5)], and selected ratios of two metabolites determined by different platforms have provided a disease-related readout [[5](#_ENREF_5)]. Metabolon and NMR data complement each other in that they cover largely different metabolite classes, and are based on different underlying techniques. Thereby, MS-based technology tends to be more sensitive, whereas NMR spectroscopy-based approaches outperform MS-based technology in terms of their reproducibility, being less susceptible to technical effects [[6](#_ENREF_6)].

20 metabolites were covered by both platforms, comprising amino acids, two fatty acids, total serum cholesterol, 3-hydroxybutyrat, glycerol, citrate, creatinine, lactate, pyruvate and urea. For these, the cross-platform correlations were computed. The median Pearson correlation coefficient was 0.72, ranging from 0.19 (linoleate) to 0.91 (3-hydroxybutyrate), and being above 0.45 for all metabolites but linoleate (Table S2 in Additional file 2, cross-platform scatter plots of metabolite concentrations are provided in Figure S2 in Additional file 3). These cross-platform correlation coefficients are within the range of those observed by Suhre et al. [[5](#_ENREF_5)] between Metabolon metabolomics measurements and measurements from a different NMR spectroscopy-based platform. Possible reasons for moderate cross-platform correlations might be found in the fact that MS and NMR spectroscopy platforms differ strongly in the underlying analytical principle and in their susceptibility to disturbing influences during sample preparation and processing. For instance, variation in pH largely influences 1H NMR spectra [[7](#_ENREF_7)] while in non-targeted MS-based methods the co-occurrence of specific metabolites in a complex mixture can affect their ionization and, thus, their quantification (ion suppression). Moderate (linear) correlation between measures from the two platforms can also be caused by differences in the quantification capabilities of the two methods: Whereas NMR spectroscopy allows for absolute quantification, the non-targeted MS-based platform only provides relative quantification and does not guarantee linearity of reported ion counts and actual concentrations of a metabolite in the samples [[6](#_ENREF_6)]. In addition, the higher sensitivity of MS-based methods at low metabolite concentrations compared to NMR spectroscopy-based platforms [[8](#_ENREF_8)] could also affect the cross-platform correlation, which is determined for the complete range of concentrations. Finally, measurement inaccuracies of both technologies might add up, also contributing to imperfect cross-platform correlations. However, disentangling the precise reasons for moderate cross-platform correlation observed for specific metabolites is outside the scope of this study, and cannot be achieved without having access to propriety information of the commercial company Metabolon.

Due to the complementarity of the two platforms and the difficulty to decide which platform provides the more meaningful measurements for a particular metabolite, we included both measurements in our analysis if a metabolite was detected on both platforms.

**Missing data handling**

**Imputation strategy**

The majority of the employed statistical methods, including linear regression and weighted correlation network analysis, require a complete data matrix. However, data from both metabolomics platforms contained a large number of missing values, arising from either technical reasons or concentrations below the limit of detection. The latter values are not missing completely at random (MCAR), i.e., the missingness of these values is not completely independent of the missing values of any other values in the data set. Given there are sufficient correlations among the variables, their missingness might be explainable by other variables in the data set, rendering them missing at random (MAR), i.e. not dependent on the missing values themselves given the observed values. Thus, we closely examined the correlation structure among the variables in our data set.

If MCAR is not given and the number of missing values is considerable, standard procedures such as complete-case analysis (i.e. dropping the missings from the analysis on a per-metabolite base) and single imputation (i.e. the generation of a single complete data set by filling up the missing values through, e.g., regression techniques) might lead to serious bias in the estimated effects and p-values [[9](#_ENREF_9)]. Consequently, multiple imputation, e.g. using multiple imputation by chained equations (MICE) [[10](#_ENREF_10)] is often a more valid strategy. MICE provides valid results if the MAR assumption is plausible, and might give a good approximation in MNAR situations [[11](#_ENREF_11)].

**Description of missingness and correlation structure**

The data set comprised 1631 observations of 582 variables (27 phenotypes, 436 Metabolon metabolites and 119 NMR metabolites after removal of metabolites representing sums, differences or ratios of other metabolites). In total, 68458 values (7.2%) were missing. The median number of missing entries among the observations was 40 (6.9%), ranging from 11 (1.9%) to 83 (13.3%). Phenotypes had at most 8 (0.5%) missing values. Of the Metabolon variables, 19 (4.4%) were completely observed, while the remaining 417 (95.6%) had missing entries. Among these, 23 variables had more than 40% missing values. The median number of missing observations was 25 (1.5%), ranging from 0 to 815 (50.0%). Of the NMR variables, 7 (5.9%) were completely observed. The median number of missing entries was 6 (0.4%), ranging from 0 to 517 (32.3%). The overall missingness pattern is visualized in Figure S3. The missingness pattern is unstructured, indicating that missingness did not co-occur in large blocks of variables, which is beneficial to the imputation process in that for the missing values of a specific variable, values of correlated variables have been observed and can be used to improve imputation of the missing values.

For the 184 variables (177 Metabolon and 7 NMR metabolites) with more than 5% missing entries, more detailed descriptive analyses were performed. First, correlation of these variables with all other variables was visualized in heatmaps to get an impression of how much information for their imputation could be borrowed from other variables (shown for the selection of 39 identified metabolites with more than 20% missings in Figure S4). Since few categorical variables were included in the data set, Kendall’s rank correlation coefficients (τ) were used. Each of the 184 variables showed absolute correlation of |τ| > 0.1 with at least one other variable in the data set (exactly one for the Metabolon metabolites leucylleucine, thymol sulfate and X-12443, and up to 187 for 1-palmitoylglycerol). On the other hand, 561 of the 582 variables in the data set provided information for at least one of the 184 variables. The strongest correlations were observed within rather than between the two metabolomics platforms.

Second, to explore the MAR assumption, correlation heatmaps of missingness indicators of the 184 variables with values of all variables were drawn (shown for the selection of 39 identified metabolites with more than 20% missings in Figure S5). Specifically for 4 NMR metabolites, XXL_VLDL_P, XXL_VLDL_PL, XXL_VLDL_L and XXL_VLDL_TG, missingness showed strong negative and positive correlations with VLDL (up to τ = -0.52) and HDL (up to τ = 0.31) metabolites, respectively. Missingness of Metabolon metabolites showed less pronounced correlations with variable values. Together, these results showed that for the majority of metabolites, the MAR assumption seemed plausible.

**Imputation settings**

Prior to imputation, the distribution of the continuous variables was investigated. Raw, natural log transformed, cubic root and square root transformed variables were tested for normality using Shapiro-Wilk tests. The transformation that showed the smallest deviation from normality was chosen (Table S9), and also kept for all subsequent statistical analyses. 350, 123 and 49 variables were log, cubic root and square root transformed, respectively, and 16 variables were not transformed.

As imputation models, predictive mean matching (PMM) and Bayesian linear regression were applied for continuous variables, and (generalized) logistic regression for binary and categorical variables. Bayesian linear regression was used for the majority of metabolites [[12](#_ENREF_12)]. Briefly, imputed values are drawn from a multivariate normal distribution defining their posterior predictive distribution given the observed values and the model parameters. Bayesian regression has the disadvantage that it relies on the normal distribution assumption of the variables to be imputed. Therefore, an extension, predictive mean matching (PMM), can be used, where the drawn values are replaced by close observed values [[9](#_ENREF_9)]. This strategy is more robust to departures from the normality assumption, and ensures that all imputed values are plausible, since they can only take observed values. This might however be undesirable in the case of MAR or MNAR values arising from values below the detection limit (i.e. actually plausible low values will be imputed with higher values). Thus, PMM was only applied to the phenotypes and to two metabolites (XXL_VLDL_P and X-12544) showing strongly asymmetric distributions even after transformation, whereas Bayesian linear regression was applied to the remaining metabolites. To avoid the occurrence of negative metabolite values generated through Bayesian linear regression, the *squeeze* function as a postprocessing step wherever variables were not log-transformed. Dichotomous and categorical variables were imputed using logistic and generalized logistic regression, respectively.

As recommended by van Buuren *et al.* [[10](#_ENREF_10)], we chose the covariates for the imputation models as follows: (1) All variables were included that were part of any of the subsequent statistical models, i.e. all phenotypic variables, including selected interactions representing later subgroup analyses. (2) In addition, for each incomplete variable, auxiliary variables were included as covariates if they correlated with the value or missingness of the respective variable at |τ| > 0.1 and were observed for at least 20% of the subjects missing the incomplete variable. The number of auxiliary variables was restricted to 30. Unidentified Metabolon metabolites were only imputed, if they represented auxiliary variables for identified variables, or for unidentified auxiliary variables. After imputation, unidentified metabolites were removed from the data set.

**Imputation diagnostics**

Convergence of the imputation algorithm was monitored by plotting mean and standard deviation of the imputed values of each variable against iteration number [[10](#_ENREF_10)]. Therefore, 5 imputations with 100 iterations each were generated. Good convergence was observed for all variables after 5 to 10 iterations (shown for the variable with largest number of missing values in Figure S6). Consequently, 10 iterations were chosen for imputation. Second, distributions of imputed and observed values of each variable were compared by means of kernel density plots (shown for two selected variables in Figure S7), revealing, as expected, by trend lower imputed than observed values for a number of metabolites (as in the example of 1,7-dimethylurate [M]).

**Combination of single imputation estimates and number of imputations**

After imputation, statistical models of interest (linear models) were fitted to each of the imputed data sets and results were summarized using the combination rules proposed by [[12](#_ENREF_12)]. The fraction of missing information γ was computed as ((1+1/M)*B)/T, where M, B and T represent the number of imputations, the between-imputation variance and the total variance of the estimate of interest, respectively.

Generally, the larger the number of imputations, the greater the precision of the estimation, owing to a smaller total variance of the estimates. The relative efficiency (RE), estimated as 1/(1+ γ/M)), describes the proportion of T that could not have been avoided by using an infinite number of imputation [[12](#_ENREF_12)]. We chose to use 20 imputations, associated with a RE > 0.97 for all analyses, as a trade-off between precision and computational time.

Weighted correlation network analysis (WGCNA) on metabolites was applied to each imputed data set. Since a high degree of similarity was observed between the resulting clustering solutions, we decided to work with a single clustering solution from one imputed data set, selecting the solution that assigned the majority of metabolites to a module, leaving the lowest number of metabolites unassigned.

**References**

1. Malone M: **Medications associated with weight gain.** *Ann Pharmacother* 2005, **39:**2046-2055.

2. Leslie WS, Hankey CR, Lean ME: **Weight gain as an adverse effect of some commonly prescribed drugs: a systematic review.** *QJM* 2007, **100:**395-404.

3. Golay A: **Metformin and body weight.** *Int J Obes* 2008, **32:**61-72.

4. **Third Report of the National Cholesterol Education Program (NCEP) Expert Panel on Detection, Evaluation, and Treatment of High Blood Cholesterol in Adults (Adult Treatment Panel III) Final Report.** *Circulation* 2002, **106:**3143.

5. Suhre K, Meisinger C, Doring A, Altmaier E, Belcredi P, Gieger C, Chang D, Milburn MV, Gall WE, Weinberger KM *et al*: **Metabolic footprint of diabetes: a multiplatform metabolomics study in an epidemiological setting.** *PLoS One* 2010, **5:**e13953.

6. Suhre K, Gieger C: **Genetic variation in metabolic phenotypes: study designs and applications.** *Nat Rev Genet* 2012, **13:**759-769.

7. Dumas ME, Maibaum EC, Teague C, Ueshima H, Zhou B, Lindon JC, Nicholson JK, Stamler J, Elliott P, Chan Q *et al*: **Assessment of analytical reproducibility of 1H NMR spectroscopy based metabonomics for large-scale epidemiological research: the INTERMAP Study.** *Anal Chem* 2006, **78:**2199-2208.

8. Barding GA, Jr., Beni S, Fukao T, Bailey-Serres J, Larive CK: **Comparison of GC-MS and NMR for metabolite profiling of rice subjected to submergence stress.** *J Proteome Res* 2013, **12:**898-909.

9. Little RJA, Rubin DB: *Statistical Analysis with Missing Data*. New York, NY, USA: John Wiley & Sons; 2002.

10. van Buuren S, Groothuis-Oudshoorn K: **mice: Multivariate Imputation by Chained Equations in R.** *Journal of Statistical Software* 2011, **45**.

11. Rässler S, Rubin DB, Zell ER: **19 Incomplete Data in Epidemiology and Medical Statistics.** *Handbook of Statistics* 2007, **27:**569-601.

12. Rubin DB: *Multiple Imputation for Nonresponse in Surveys*. New York, NY, USA: John Wiley & Sons; 1987.
