# Supplementary material for: Multi-omic signature of body weight change: results from a population-based cohort study
Source: BMC Med. 2015 Mar 9;13:48. doi: 10.1186/s12916-015-0282-y (PMC4367822; doi:10.1186/s12916-015-0282-y)
Supplement: Additional file 3: Figure S1. — Distribution of the variability of the day medians across the Metabolon metabolites. Figure S2. Scatter plot of serum measurements on the Metabolon and NMR platforms. Figure S3. Missingness pattern in the metabolomics data set. Figure S4. Correlation among metabolites and phenotypes. Figure S5. Correlations of missingness with variable values. Figure S6. Convergence of the MICE algorithm for the metabolite with the largest proportion of missings (3-hydroxy-2-ethylpropionate [M]). Figure S7. Imputation diagnostics for two selected variables. Figure S8. Choice of the scale-free topology parameter in weighted correlation network analysis (WGCNA). Figure S9. Weighted correlation network analysis (WGCNA) cluster dendrograms. Figure S10. Joint coverage of the serum metabolome by the four metabolite modules related to annual percentage body weight change (ΔBW). Figure S11. Association of annual percentage body weight change (ΔBW) with lipoprotein subclasses. Figure S12. Multi-omic partial correlation network comprising the 8 metabolite and the 19 gene expression modules. Figure S13. Association of annual percentage body weight change (ΔBW) with members of associated metabolite modules (MetM) – results of the stratified analyses. Figure S14. Association of annual percentage body weight change (ΔBW) with omics modules adjusting for factors driving weight change. [file 12916_2015_282_MOESM3_ESM.pdf]

Multi-omic signature of body weight change: results from a population-based cohort study

Simone Wahl*, Susanne Vogt*, Ferdinand Stückler, Jan Krumsiek, Jörg Bartel, Tim Kacprowski, Katharina Schramm, Maren Carstensen, Wolfgang Rathmann, Michael Roden, Carolin Jourdan, Antti J Kangas, Pasi Soininen, Mika Ala-Korpela, Ute Nöthlings, Heiner Boeing, Fabian J Theis, Christa Meisinger, Melanie Waldenberger, Karsten Suhre, Georg Homuth, Christian Gieger, Gabi Kastenmüller, Thomas Illig, Jakob Linseisen, Annette Peters, Holger Prokisch, Christian Herder, Barbara Thorand#, Harald Grallert#

Additional file 3: Supplementary Figures

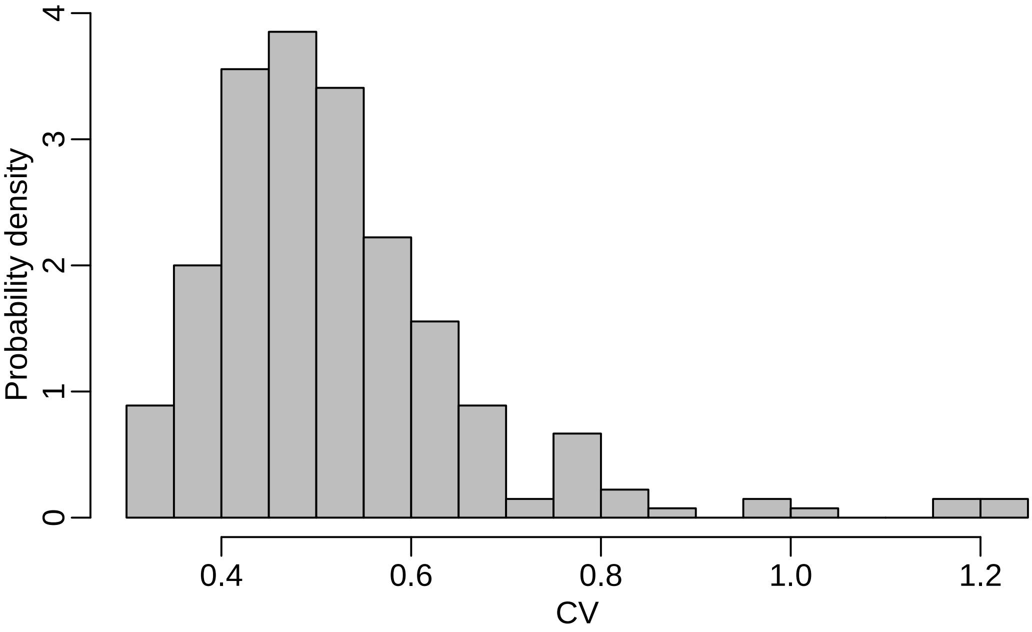

Figure S1: Distribution of the variability of the day medians across the Metabolon metabolites. Variability is expressed as coefficient of variation (CV) = standard deviation devided by mean.

*,# contributed equally

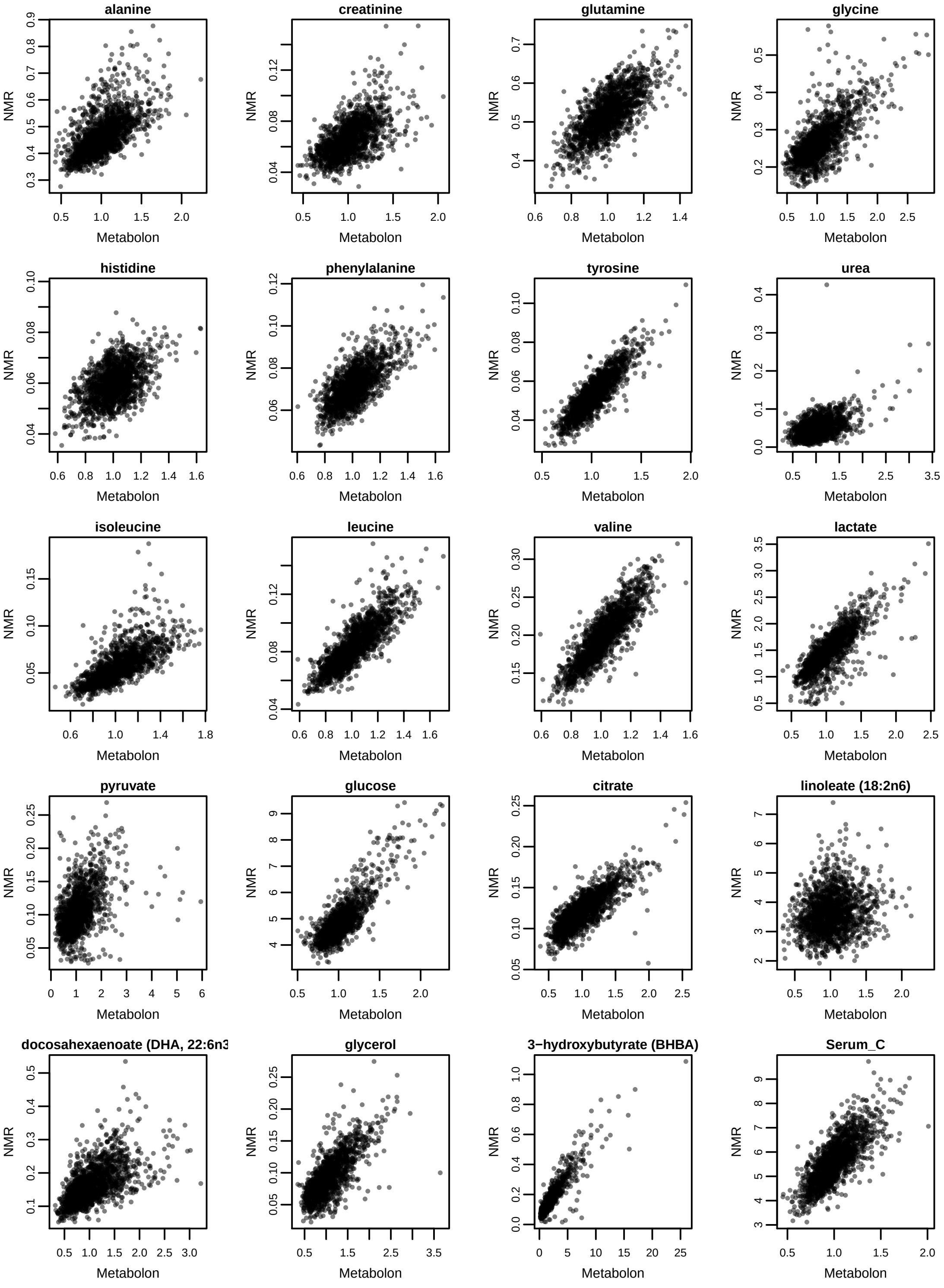

Figure S2: Scatter plot of serum measurements on the Metabolon and NMR platforms. Concentrations are shown in mmol/L.

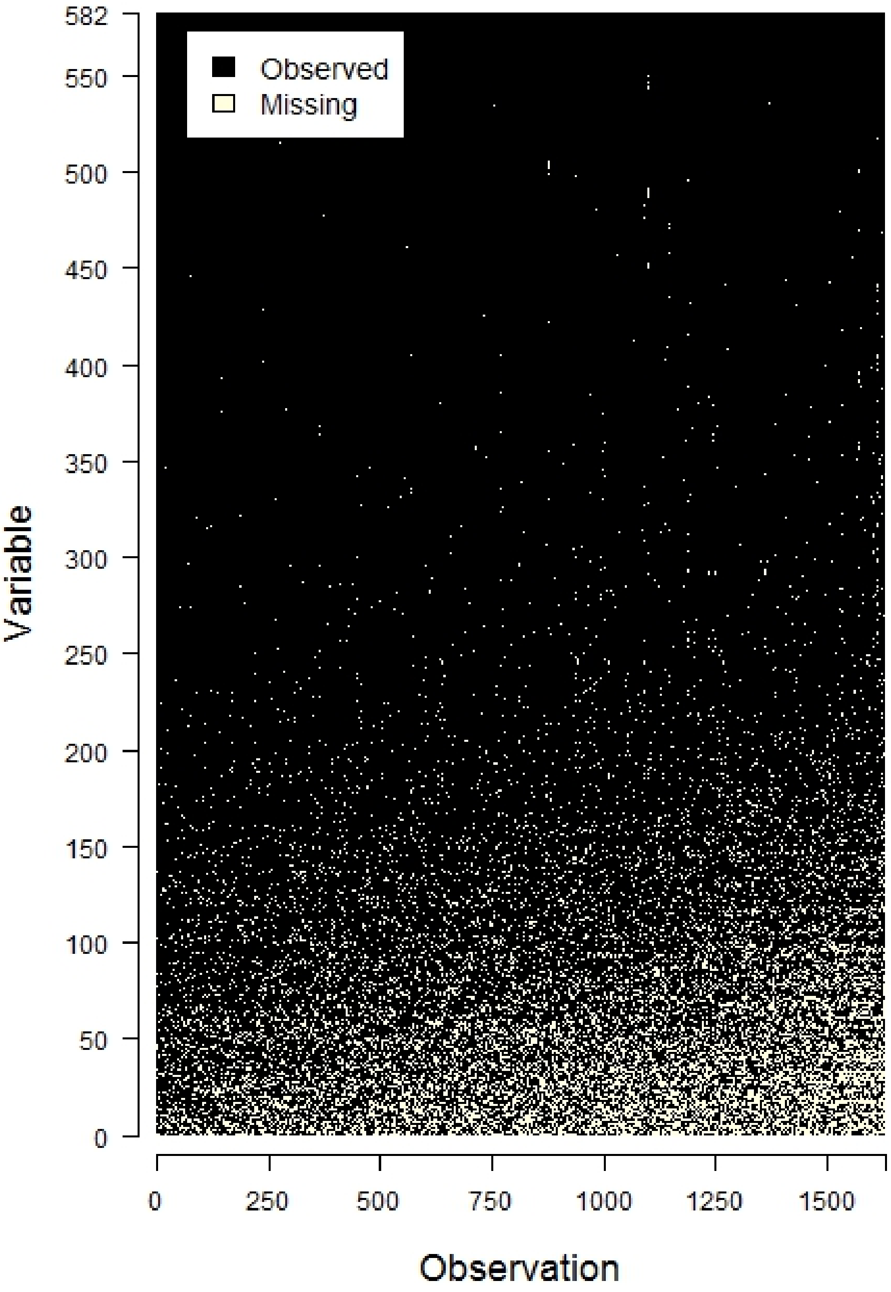

Figure S3: Missingness pattern in the metabolomics data set. Plot of missingness indicators (black, observed; light yellow, missing) for the 582 variables against the 1631 observations, both sorted by percentage of missing values.

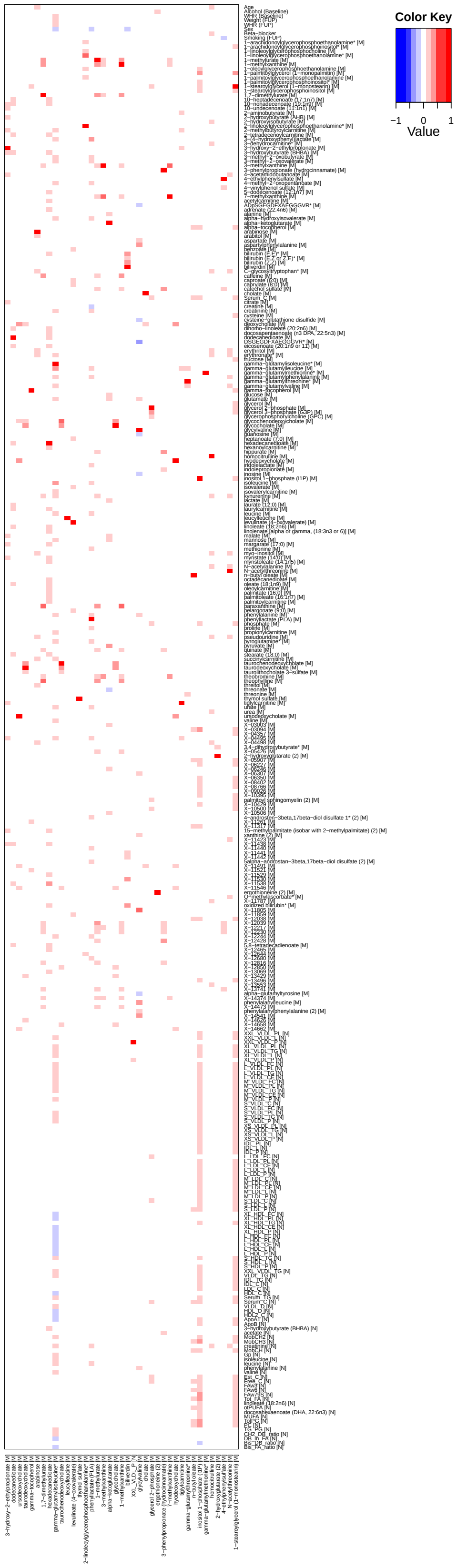

Figure S4: Correlation among metabolites and phenotypes. Heatmap of Kendall's correlation coefficient τ of the 39 identified metabolites with at least 20% missing values (columns; sorted from left to right by decreasing proportion of missings) against the 257 variables correlated with at least one of the 39 metabolites at $|\tau| \geq 0.15$ (rows). White, $|\tau| < 0.15$; red, $\tau \geq 0.15$; blue, $\tau \leq 0.15$.

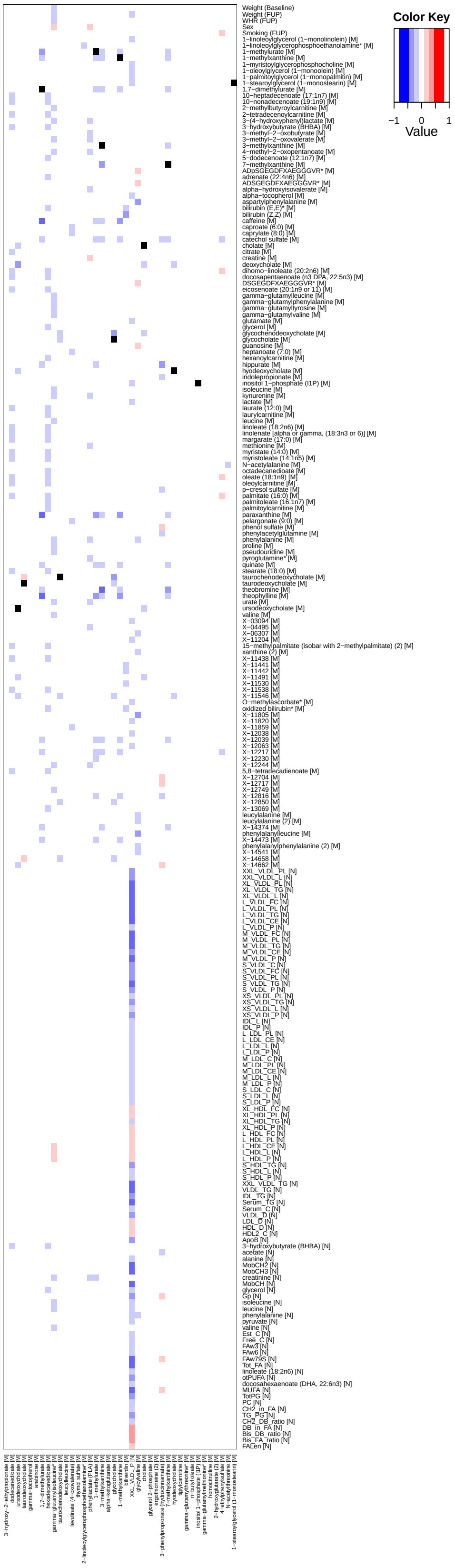

Figure S5: Correlation among metabolites and phenotypes. Heatmap of Kendall's correlation coefficient τ of the missingness indicator (0, observed; 1, missing) of the 39 identified metabolites with at least 20% missing values (columns; sorted from left to right by decreasing proportion of missings) against the 328 variables correlated with missingness of at least one of the 39 metabolites at $|\tau| \geq 0.15$ (rows). White, $|\tau| < 0.15$; red, $\tau \geq 0.15$; blue, $\tau \leq 0.15$.

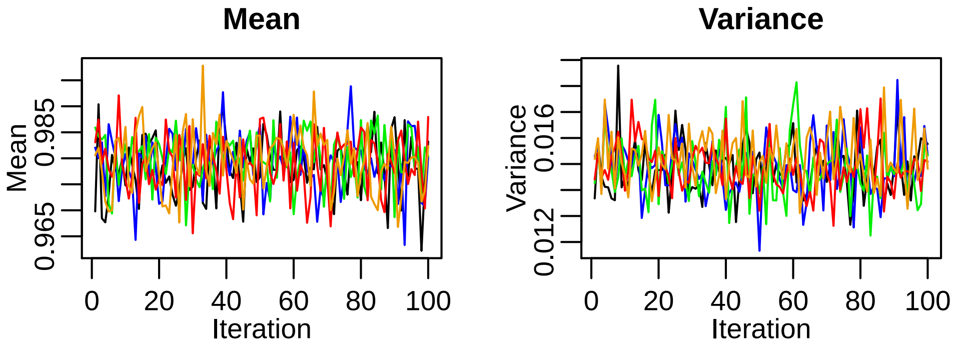

Figure S6: Convergence of the MICE algorithm for the metabolite with the largest proportion of missings (3-hydroxy-2-ethylpropionate [M]). Plotted is mean (left) and variance (right) of imputed values for each of 5 imputation chains across 100 iterations.

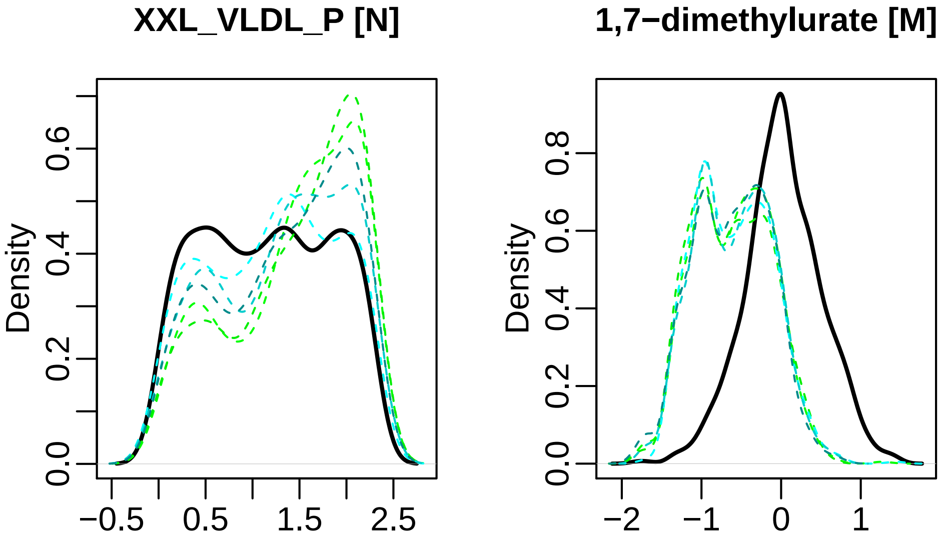

Figure S7: Imputation diagnostics for two selected variables. Kernel density plots of observed (black solid line) vs. imputed (dashed lines in different shades of green) values, shown for the first five imputations.

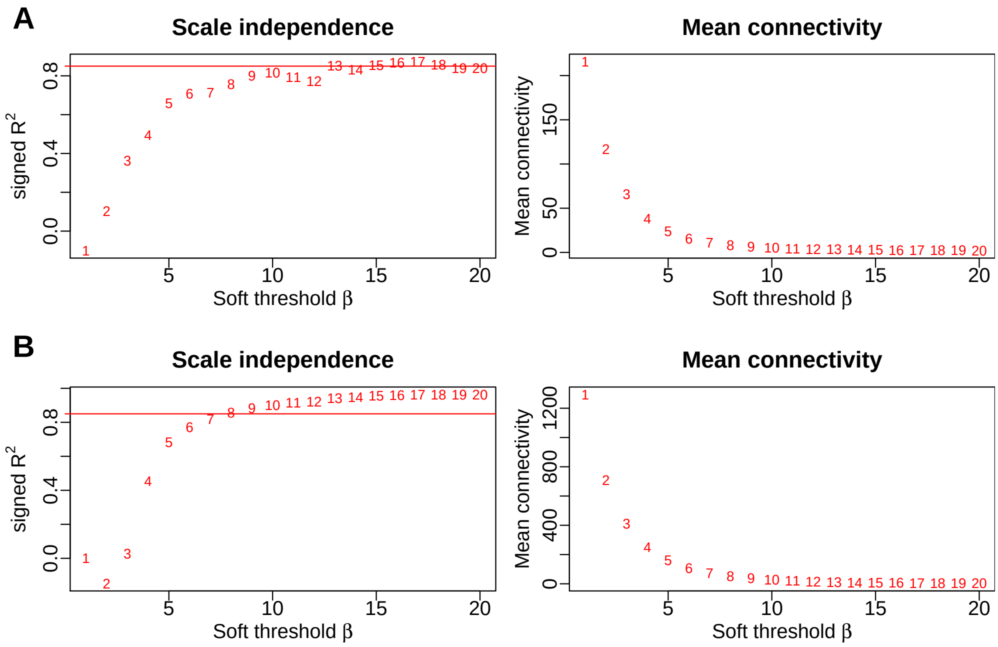

Figure S8: Choice of the scale-free topology parameter β in weighted correlation network analysis (WGCNA). A Metabolomics data. **B** Gene expression data. The smallest β (13 and 8, respectively) with $R^2 \geq 0.85$ was chosen.

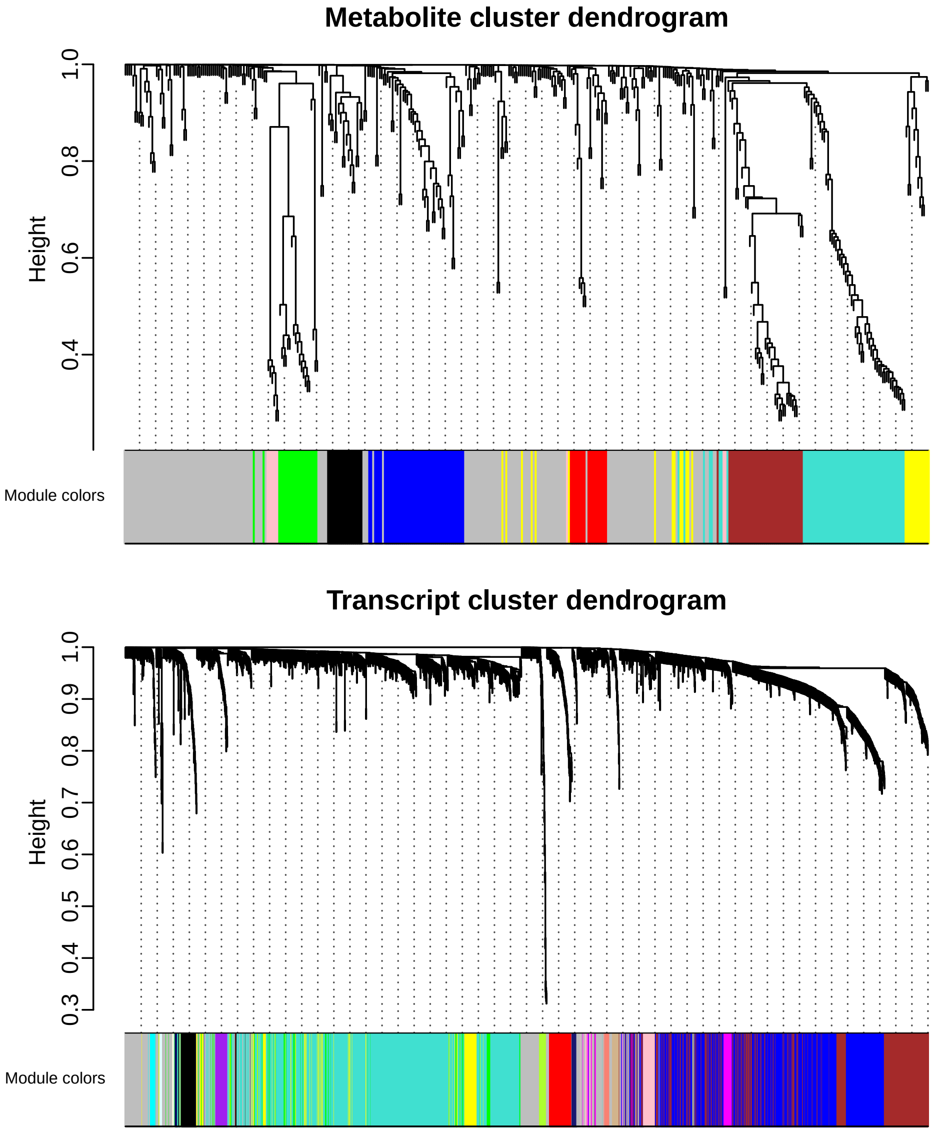

Figure S9: Weighted correlation network analysis (WGCNA) cluster dendrograms. Metabolomics data and gene expression data were clustered into 8 and 19 modules, respectively. Grey color represents features showing a low connectivity that are not assigned to a cluster.

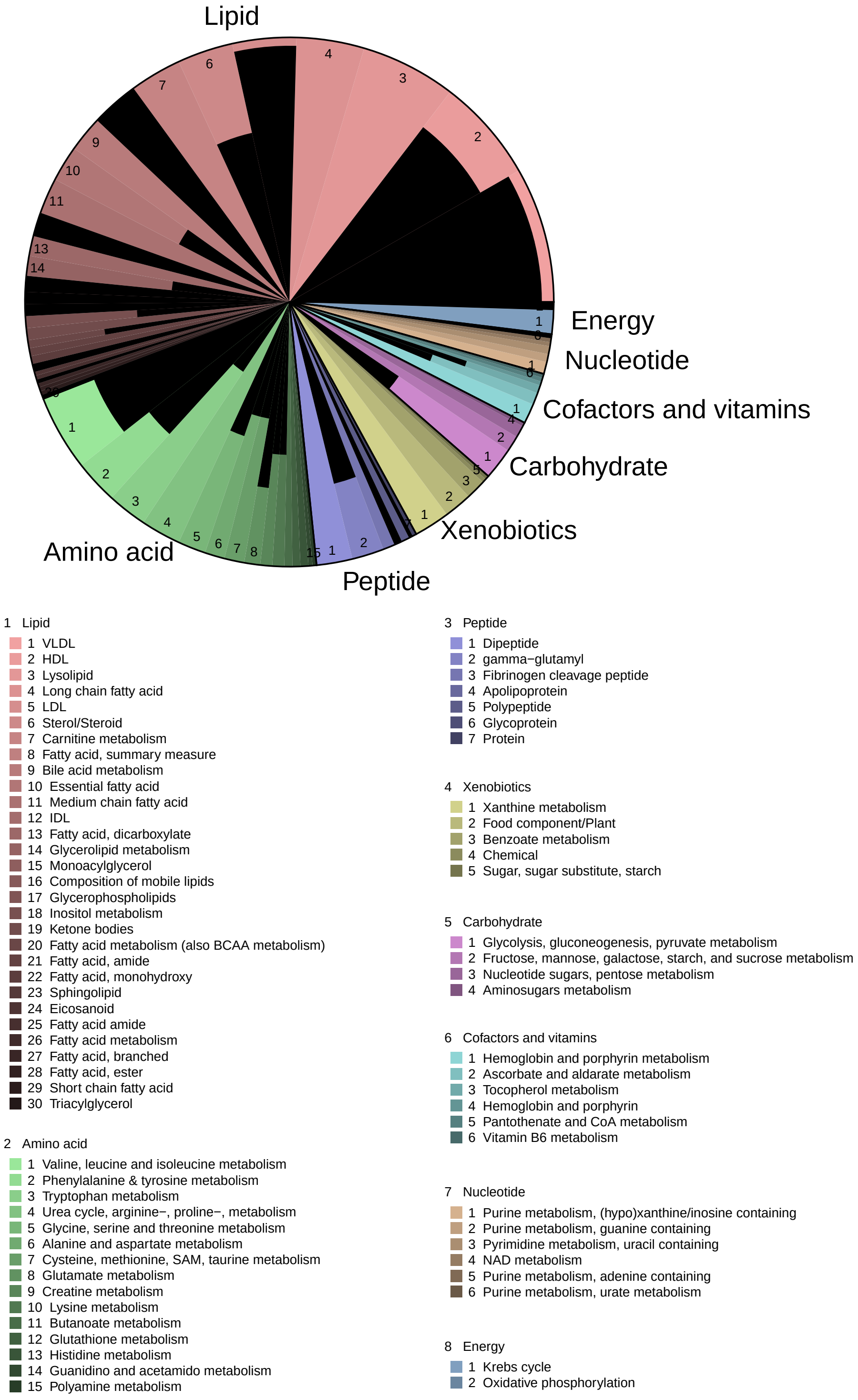

Figure S10: Joint coverage of the serum metabolome by the four metabolite modules related to annual percentage body weight change (ΔBW). Pie chart with color indicating super-/sub-pathway as described in the legend, and size of wedges representing the number of metabolites in the data set corresponding to the respective sub-pathway. Sorted by pathway size. Black wedges represent the joint number of metabolites from MetM1 (the TG/VLDL module), MetM3 (the LDL/IDL module), MetM4 (the BCAA module) and MetM5 (the HDL module) in the respective sub-pathway.

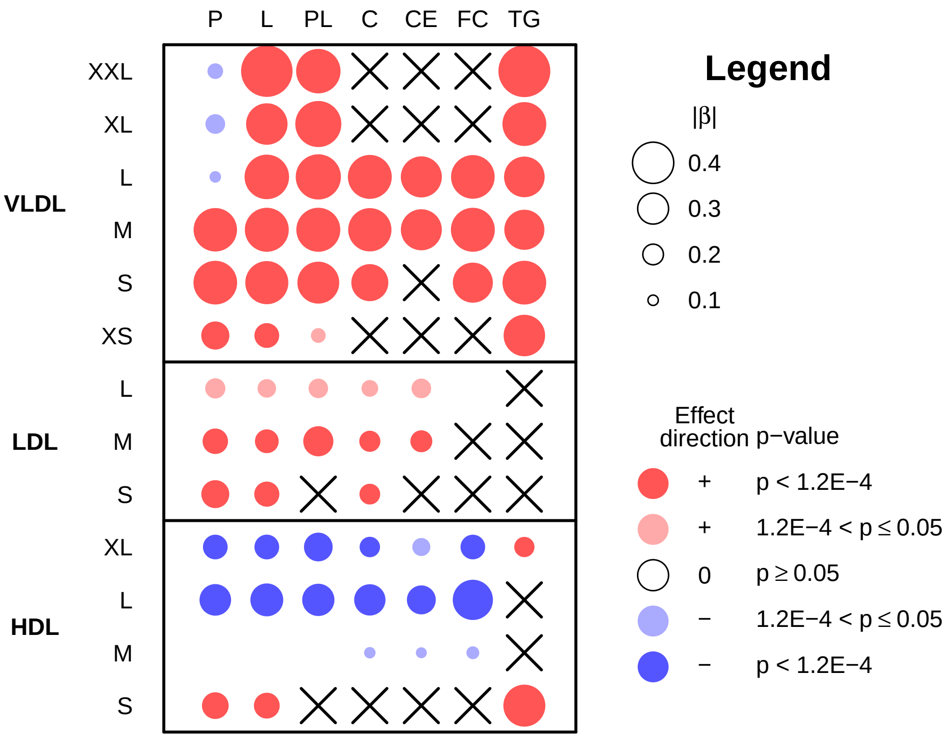

Figure S11: Association of annual percentage body weight change (ΔBW) with lipoprotein subclasses. Bubbles represent effects strengths and significance, as described in the legend. Models were adjusted for age, sex and baseline body weight. For single metabolites, the significance threshold was chosen as $P < 1.2 \times 10^{-4}$ corresponding to Bonferroni correction for 441 tests. P, particle concentration; L, total lipids; PL, phospholipids; C, total cholesterol; CE, cholesterol esters; FC, free cholesterol; TG, triglycerides. Note that C represents the sum of CE and FC, and L the sum of C, PL and TG.

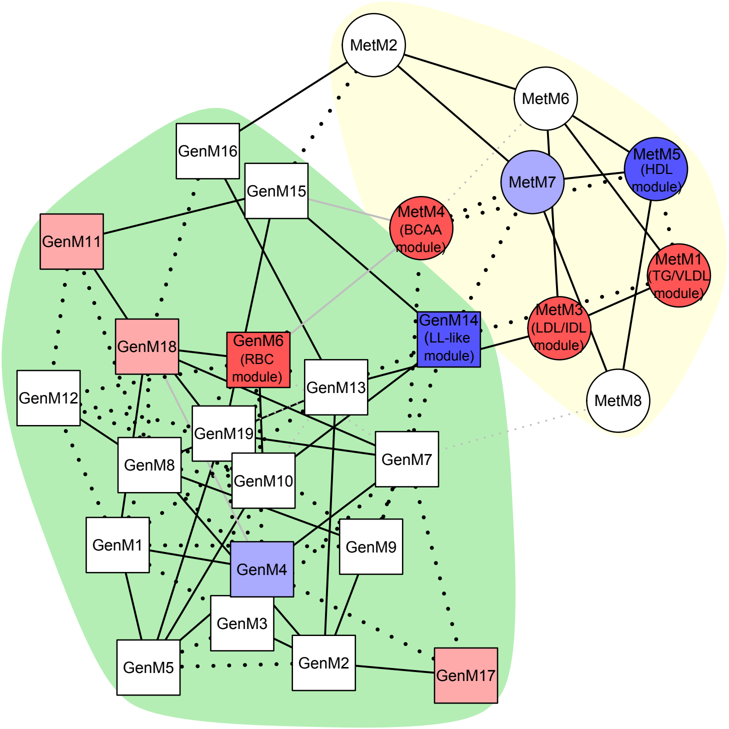

Figure S12: Multi-omic partial correlation network comprising the 8 metabolite and the 19 gene expression modules. Nodes represent omics modules (circle, metabolite module (MetM); rectangle, gene expression module (GenM)), colored according to their association with annual percentage body weigth change (ΔBW; red, positive association; blue, negative association; bright color, significant $P < 1.9 \times 10^{-4}$; light color, $P < 0.05$). Edges represent partial correlations (ζ) between pairs of modules (represented by their module eigengenes (MEs)), conditional on all other presented modules and the covariates age, sex, and ΔBW (solid black line, $\zeta > 0.1$; dotted black line, $\zeta < -0.1$; solid grey line, $0.05 < \zeta < 0.1$; dotted grey line, $-0.1 < \zeta < -0.05$). Background color reflects metabolite (yellow) vs. gene expression (green) modules.

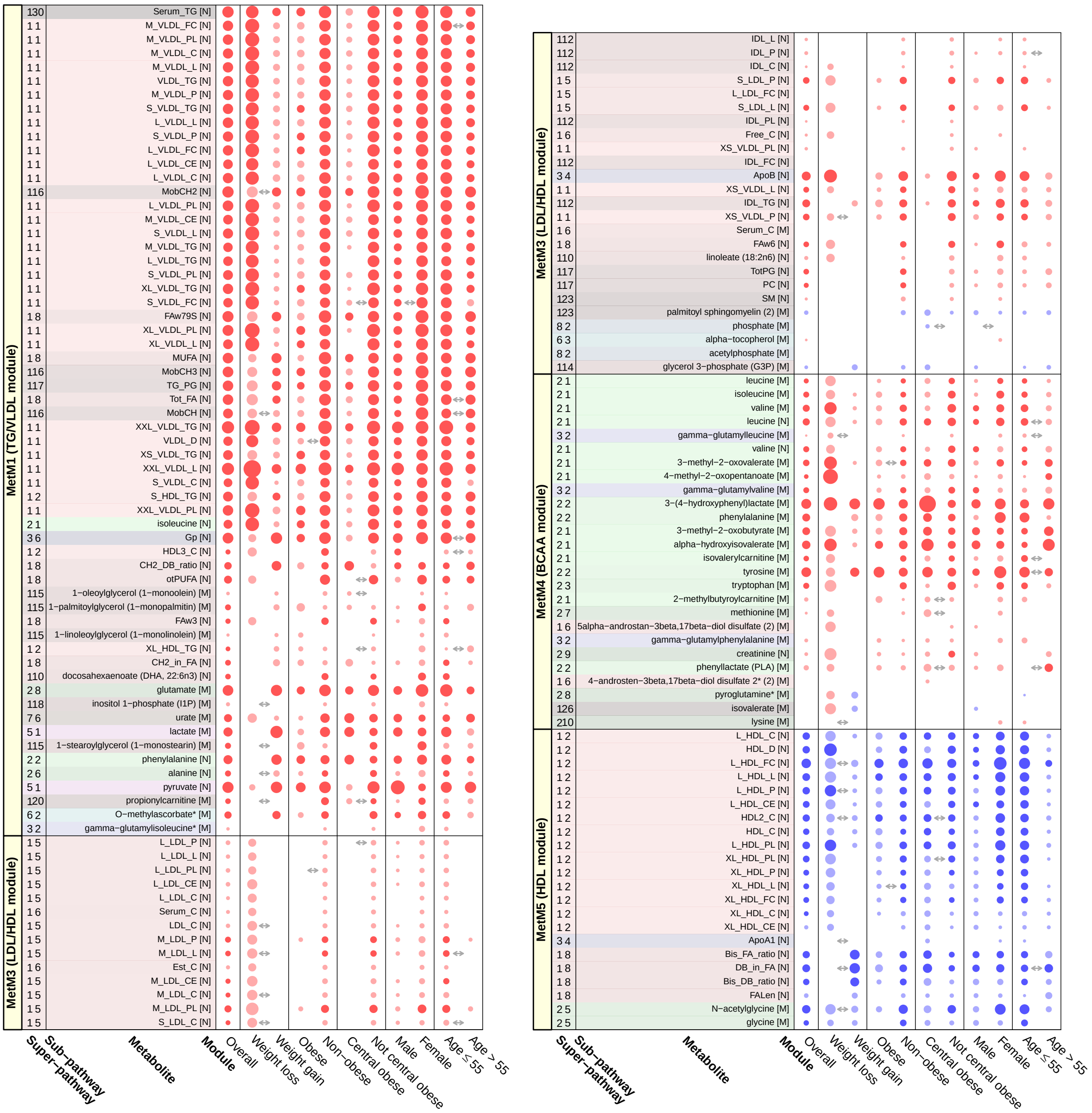

Figure S13: Association of annual percentage body weight change (ΔBW) with members of associated metabolite modules (MetM) – results of the stratified analyses. Shown are associations for the overall study population (column 1) and for subgroups (columns 2-11). Bubbles represent effect strengths and significance, see legend of Figure 2. Models were adjusted for age, sex and baseline body weight. Significance threshold $P < 1.2 \times 10^{-4}$ corresponds to Bonferroni correction for 411 metabolites. For subgroup analyses (columns 2-11), interaction models were fitted, to obtain main ΔBW effect in the respective subgroups, and ΔBW:subgroup interaction effect indicating difference in effect between the subgroups. Background colors correspond to super- and sub-pathway annotation, see legend of Figure S8 in Additional file 3.

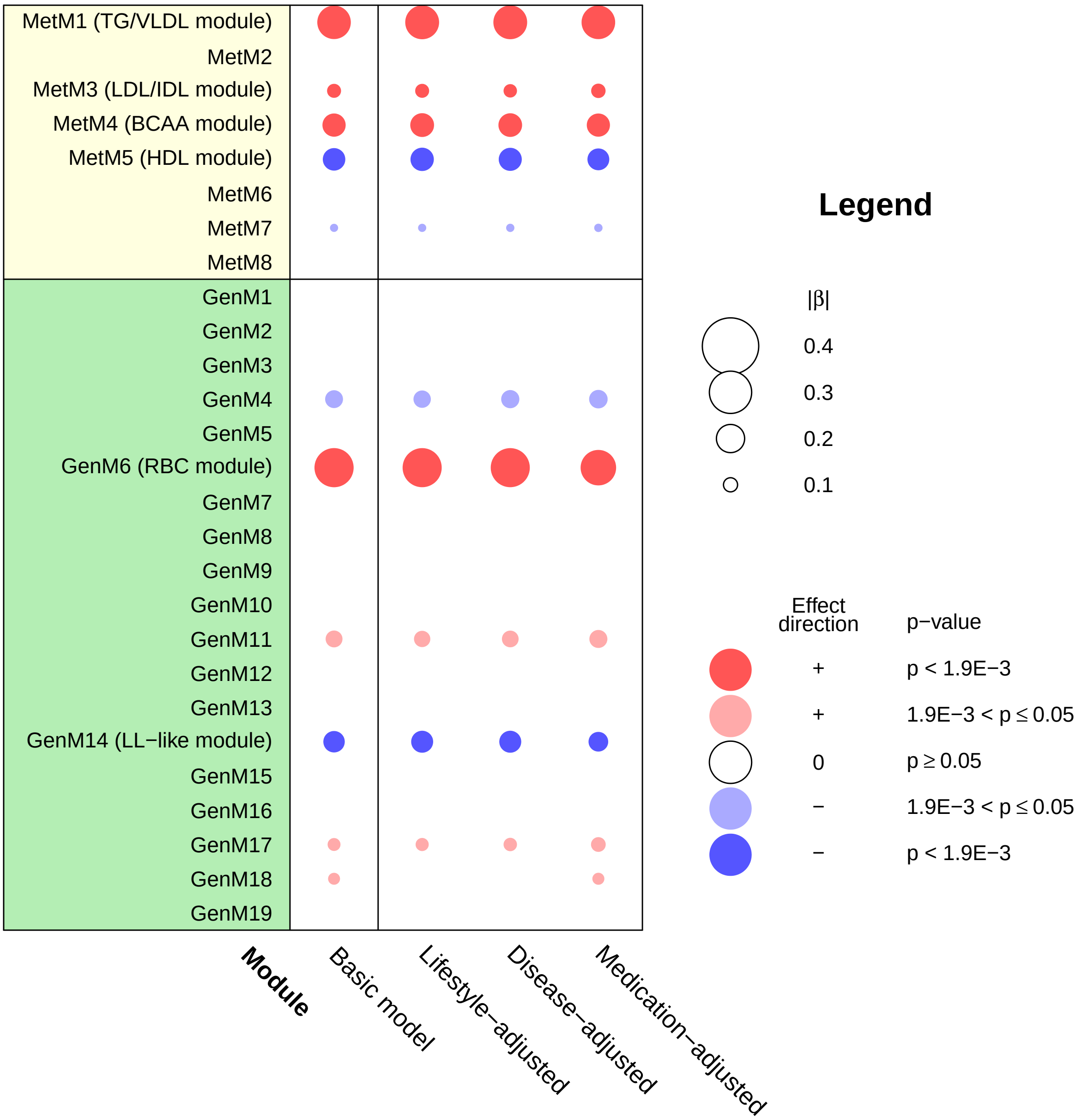

Figure S14: Association of annual percentage body weight change (ΔBW) with omics modules adjusting for factors driving weight change. Bubbles represent effect strengths and significance, as described in the legend. Models were adjusted for age, sex and baseline body weight. Significance threshold $P < 1.9 \times 10^{-3}$ corresponds to Bonferroni correction for 27 modules. Lifestyle-adjusted: changes in physical activity, baseline nutritional score, changes in sleeping behavior, smoking and alcohol drinking were included as covariates in the model. Disease-adjusted: incident diabetes, cancer, myocardial infarction and stroke were included as covariates in the model. Medication-adjusted: change in the intake of beta-blockers, metformin, other antidiabetic drugs, systemic corticoids, oral contraceptives and antidepressants were included as covariates in the models (see descriptives in Table S9). GenM, gene expression module; MetM, metabolite module.
